# Tuning Isomerism Effect in Organic Bulk Additives Enables Efficient and Stable Perovskite Solar Cells

**DOI:** 10.1007/s40820-024-01613-z

**Published:** 2025-01-10

**Authors:** Qi Zhang, Qiangqiang Zhao, Han Wang, Yiguo Yao, Lei Li, Yulin Wei, Ruida Xu, Chenyang Zhang, Erik O. Shalenov, Yongguang Tu, Kai Wang, Mingjia Xiao

**Affiliations:** 1 Institute of Flexible Electronics (IFE), Northwestern Polytechnical University (NPU), Xi’an, 710072 People’s Republic of China; 2 School of Management, Xián Polytechnic University, Xi’an, 710072 People’s Republic of China; 3 The Quzhou Affiliated Hospital of Wenzhou Medical University, Quzhou People’s Hospital, Quzhou, 324000 People’s Republic of China; 4 Institute of Experimental and Theoretical Physics, Al-Farabi Kazakh National University, Almaty, 050040 Kazakhstan

**Keywords:** Organic additives, Molecular simulation, Perovskite solar cells, Passivation, Isomeric effect

## Abstract

Organic additives with multiple functional groups have shown great promise in improving the performance and stability of perovskite solar cells. The functional groups can passivate undercoordinated ions to reduce nonradiative recombination losses. However, how these groups synergistically affect the enhancement beyond passivation is still unclear. Specifically, isomeric molecules with different substitution patterns or molecular shapes remain elusive in designing new organic additives. Here, we report two isomeric carbazolyl bisphosphonate additives, 2,7-CzBP and 3,6-CzBP. The isomerism effect on passivation and charge transport process was studied. The two molecules have similar passivation effects through multiple interactions, e.g., P = O···Pb, P = O···H–N and N–H···I. 2,7-CzBP can further bridge the perovskite crystallites to facilitates charge transport. Power conversion efficiencies (PCEs) of 25.88% and 21.04% were achieved for 0.09 cm^2^ devices and 14 cm^2^ modules after 2,7-CzBP treatment, respectively. The devices exhibited enhanced operational stability maintaining 95% of initial PCE after 1000 h of continuous maximum power point tracking. This study of isomerism effect hints at the importance of tuning substitution positions and molecular shapes for organic additives, which paves the way for innovation of next-generation multifunctional aromatic additives.

**Supplementary Information:**

The online version contains supplementary material available at 10.1007/s40820-024-01613-z.

## Highlights

- By anchoring the perovskite sites with the functional groups of CzBP (P = O···Pb, N–H···I and P = O···N–H), the bulk nonradiative recombination is suppressed and ion migration is inhibited. Doping perovskite films with CzBP led to enhanced intercrystallite interactions in the bulk and improved photoluminescence quantum yield.
- Using a typical electron-rich moiety as the π-linker to replace the classic alkyl spacer in CzBP facilitated the charge-carrier transport processes and the passivation effect of carbazole further contributed to high *V*_OC_. The optimized 2,7-CzBP-treated device achieves the highest power conversion efficiency (PCE) of 25.88%, with *V*_OC_ of 1.189 V for 0.090 cm^2^ and the perovskite solar cell module with a PCE of 21.04% for 14 cm^2^.
- For 2,7-CzBP, the more extended conjugation and the more linear molecular geometry result in a more effective improvement in the performance.

**Supplementary Information:**

The online version contains supplementary material available at 10.1007/s40820-024-01613-z.

## Graphical abstract

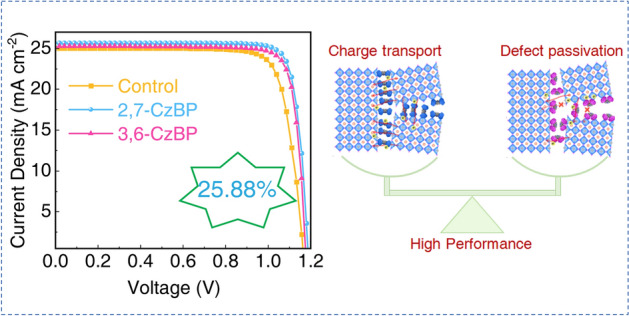

## Introduction

The power conversion efficiency (PCE) of single-junction perovskite solar cells (PSCs) has skyrocketed to > 26% in recent years [1]. However, the efficiency is still far below the Shockley–Queisser limit and the durability remains a major barrier to commercialization. Organic additives are widely used in improving efficiency and stability of PSCs by passivating defects [2–4]. These additives contain polar functional groups such as amino, hydroxy and carbonyl. Related works researched the chemical space of organic additives by permutation of these groups, yet the synergy remains unclear. Typically, most organic additives are insulating materials that may hinder charge transport, and knowledge about other effects beyond passivation is limited [5, 6]. Custom-tailored organic additives that simultaneously work as superior morphology modulators, phase stabilizers, energy-level adjusters, defect passivators and stability enhancers are rare. There is much room to unveil the mechanism and develop universal guidelines for innovation of next-generation organic additives.

Beginning in 1970s, conjugated organic materials have found extensive use in electronics and photonics. Specifically, in single-molecule electronics, conjugated molecules installed by symmetric functional groups at the terminals present a myriad of interesting electrical properties. We expect that such structural motif shows great potential in designing bulk additives for PSCs, because the conjugated units can undoubtedly facilitate charge transport. A conjugated bulk additive may function as a bridge to connect cracked crystallites, with two binding sites to passivate undercoordinated ions at PVK surface. Different substitution positions of the functional groups further allow us to study the isomerism effect on device performance. Such structure–property relationship is valuable to unveil the underlying mechanism and expand the reach of new bulk additives.

**Fig. 1:**
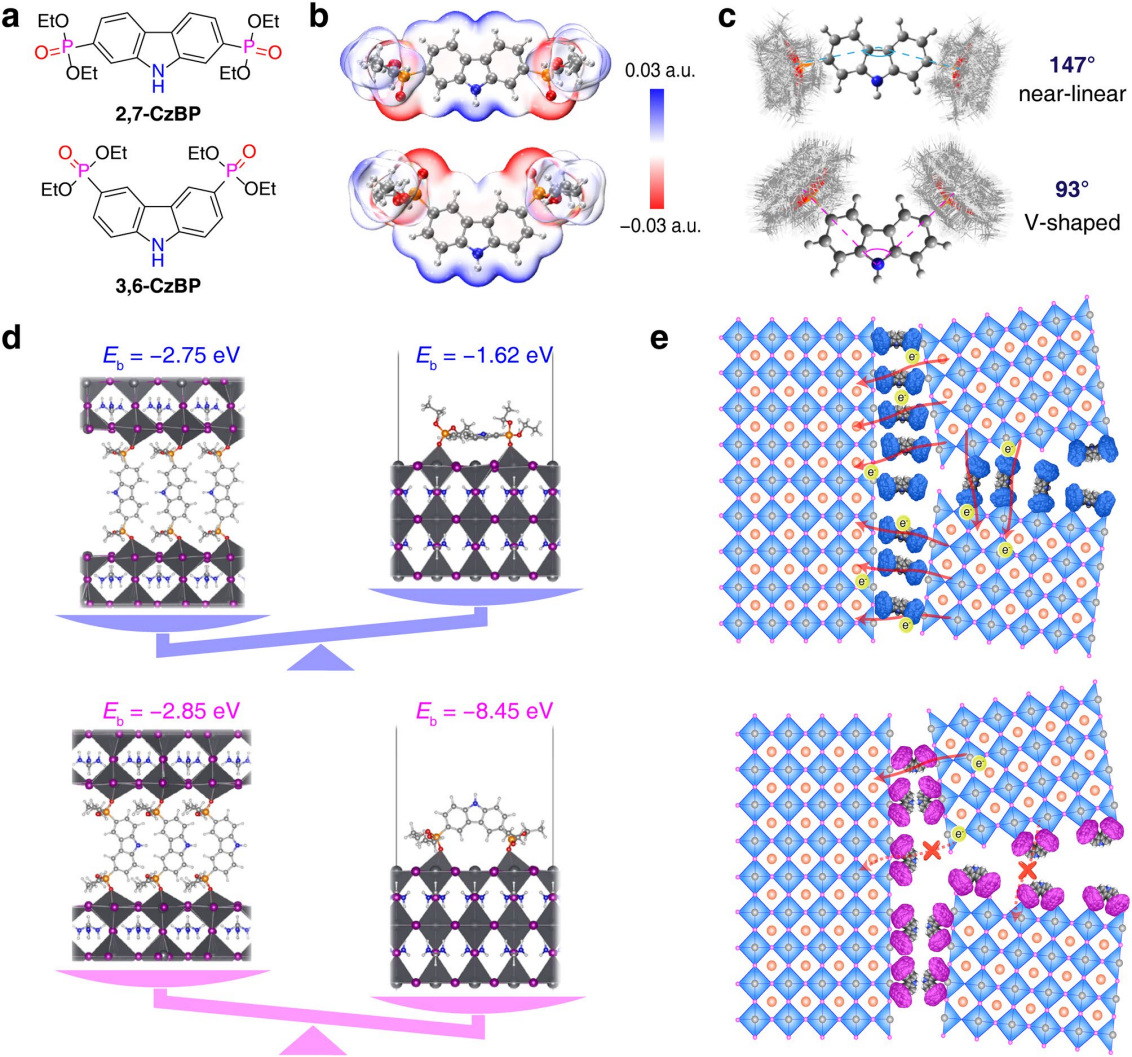
Theoretical study of 2,7-CzBP and 3,6-CzBP. **a** Molecular structures of 2,7-CzBP and 3,6-CzBP. **b** ESP isosurfaces calculated at the B3LYP-D3(BJ)/def2-SVP level. **c** Trajectories of 2,7- and 3,6-CzBP by molecular dynamics simulation. **d** Comparison of the binding energies of two bi-coordination modes. **e** Schematic charge transport paths in 2,7-CzBP and 3,6-CzBP perovskite films

With these concepts in mind, we designed and synthesized two isomeric molecules (Fig. 1a), 9*H*-carbazole-2,7-diyl tetraethyl bisphosphonate (2,7-CzBP) and 9*H*-carbazole-3,6-diyl tetraethyl bisphosphonate (3,6-CzBP). We use phosphonate esters as the binding sites and carbazole as the conjugated core [7, 8]. Organic esters do not corrode metal oxide surfaces or encounter unforeseen difficulties due to acidity during preparation, while retaining Lewis-base binding sites for passivation of undercoordinated cations [9, 10]. These features are highly compatible to one-step bulk passivation by mixing the additive and the perovskite precursors before deposition [11]. We expect that the bisphosphonate can form dual coordination to enhance intercrystallite interactions in the bulk and the carbazole π-linker can facilitate charge transport through the bridge. We studied the isomerism effect by exploring multiple interactions (e.g., P = O···Pb, P = O···H–N and N–H···I), microstructures and solar cell performances. Tuning this isomerism effect allows efficient and stable perovskite solar cells with a PCE of 25.88%. Different substitution patterns lead to distinct coordination modes, while molecular geometry impacts charge transport behaviors spanning multiple scales.

## Experimental Section

### Materials

Fluorine-doped tin oxide (FTO) glass substrates, lead iodide (PbI_2_), formamidinium iodide (FAI), 4-*tert*-butypyridine and lithium bis(trifluoromethanesulfonyl) imide (Li-TFSI), tin (IV) chloride, 2,2*’*,7,7*’*-tetrakis[*N,N*-di(4-methoxyphenyl)amino]-9,9*’*-spirobifluorene (spiro-OMeTAD) were purchased from YOUXUAN Technology Co. Ltd. (China). Dimethyl sulfoxide (DMSO), *N,N*-dimethylformamide (DMF), ethyl acetate (EA), chlorobenzene (CB) and 4-*tert*-butylpyridine (*t*BP) were purchased from Aladdin. All chemicals and reagents were used as received without any further purification.

### Device Fabrication and Characterization

#### Film Deposition and Device Fabrication

FTO glass substrates were cleaned via sequential sonication (30 min for each) with detergent in water once, deionized water twice and ethanol once. For ETL deposition, SnO_2_ precursor solution was obtained by mixing the anhydrous SnCl_4_ solution with deionized water in a volume ratio of 1:75, and then the solution was aged at 25 °C for two weeks to obtain a white colloidal solution. Then the SnO_2_-Cl layer was deposited by spin-coating at 3000 rpm for 30 s and annealed in ambient air at 180 °C for 30 min. For the fabrication of (FA_0.95_Cs_0.05_PbI_3_)_0.975_(MAPbBr_3_)_0.025_ PSCs, the perovskite solution was prepared by mixing 705.3 mg of PbI_2_, 228.8 mg of FAI, 18.2 mg of CsI, 33.7 mg of MACl and 17.2 mg of MAPbBr_3_ in a mixed solvent of 889 µL of DMF and 111 µL of DMSO. The amounts of 9*H*-carbazole-2,7-diyl tetraethyl bisphosphate (2,7-CzBP) and 9*H*-carbazole-3,6-diyl tetraethyl bisphosphate (3,6-CzBP) added were from 0.1 to 1.0 mg mL^−1^. The perovskite solution was spin-coated on the SnO_2_ substrates at 1000 rpm (500 rpm ramp) and 5000 rpm (1000 rpm ramp) for 10 and 30 s, respectively. An antisolvent of ethyl acetate (400 *μ*L) was drop-casted quickly at the last 5 s of the second step. These films were transferred onto a hotplate in ambient air with a relative humidity (RH) of < 30% and heated at 100 °C for 40 min. Later, the spiro-OMeTAD solution as hole transporting material was prepared by dissolving 72.3 mg of spiro-OMeTAD in 1.000 mL of CB and 46.3 µL of the mixed solution of 1.645 mL of tBP and 1.000 mL of Li-TSFI solution (520 mg of Li-TSFI in 1.000 mL of ACN was added). Forty microliters of spiro-OMeTAD solution was deposited on perovskite films by spin-coating (3000 rpm, 30 s). For operational testing and under conditions of high temperature and high humidity, the spiro-OMeTAD was replaced with PTAA. Finally, 60-nm Au electrodes were thermally evaporated under vacuum (< 4 × 10^−4^ Pa) to complete the device fabrication and aging devices for 20 h in a drying air box.

For fabricating modules, P1 etching process was pre-patterned on the cleaned FTO glass (5 cm × 5 cm) with a 1064 nm fiber laser (Han’s laser). Then, patterned FTO substrates were cleaned and treated by UV Ozone Cleaner (Ossila) for 15 min. The FTO substrate was pre-patterned for P1 (40 *μ*m wide) at 60% laser power and a speed of 300 mm s^−1^ with a frequency of 65 kHz and pulse width of 120 ns. The subsequent processes for the preparation of layers were prepared with the same procedure as presented above. The large-size raw (FA_0.95_Cs_0.05_PbI_3_)_0.975_(MAPbBr_3_)_0.025_ perovskite film was prepared through the doctor-blading method (provided by Suzhou GCL Nano Co. Ltd.). For P2 etching process, the laser was a 532-nm laser. 100-nm-thick Ag electrodes were thermally evaporated under a vacuum to complete the module’s fabrication. Finally, P3 etching used the same laser as P2.

#### Characterization

Fourier transform infrared (FTIR) spectra were recorded on a Nicolet iN10 spectrometer by mixing CzBP powder into potassium bromide (KBr). Perovskite film fabricated using perovskite precursors consists of 2,7-CzBP/PbI_2_ (1:1, mol%) and 2,7-CzBP/FAI (1:1, mol%) in DMF: DMSO (8:1, v/v). X-ray photoelectron spectroscopy (XPS) tests were carried out with a photoelectron spectrometer model of ESCA LAB 250Xi from Thermo Fisher Scientific in the USA (A1 monochromatic aluminum *K*α anode target, 1486.6 eV, X-ray source: 150 W, sampling depth: 1–10 nm, scanned area: 500 × 500 *μ*m^2^). The charging correction was conducted with adventitious carbon at 284.8 eV. ^1^H NMR and ^13^C NMR spectra were collected on a Bruker spectrometer in DMSO-*d*_*6*_. Top-view and cross-sectional scanning electron microscope (SEM) images were obtained with a field-emission scanning electron microscope (S–4800, Hitachi). The surface morphology of the films was tested by atomic force microscopy (AFM, Agilent 5400AFM). The crystal structure of the control and CzBP-treated films was characterized by X-ray diffraction (XRD) (Bruker-AXS Micro diffractometer) using Cu *K*α (*λ* = 0.15406 nm) source. GIWAXS data were collected with a beam energy of 10 keV. GIWAXS parameters were calibrated using a LaB_6_ standard sample. The GIWAXS pattern was converted using GIWAXS-Tools software. The optical absorbance spectra were measured by UV–Vis/NIR spectrophotometer (U-4100, Hitachi). The UPS equipped with He–I source (*hʋ* = 21.22 eV) (AXIS ULTRA DLD, Kratos, UK) was used to determine the valence band energy and the Fermi level. The Fermi level of the samples was referred to that of Au which was in electrical contact with a sample in UPS measurements. Photoluminescence (PL) spectra were recorded on a Perkin LS-55 fluorescence spectrometer excited at 450 nm (structure: glass/PVK, excitation from the film side). PL mapping measurements were taken on a Raman image-scanning electron microscope (LabRAM HR Evolution). Structure: FTO/SnO_2_/PVK with and without CzBP treatment, excitation from the film side. The time-resolved PL spectra were measured using an FLS1000 Edinburgh Instruments spectrofluorometer equipped with the integrating sphere (structure: glass/PVK, excitation from the film side). The TRPL data were modeled by a bi-exponential formula: [*I*(*t*) = *I*_0_ + *A*_1_exp(− *t*/*τ*_1_) + *A*_2_exp(− *t*/*τ*_2_)], where fast (*τ*_1_) and slow (*τ*_2_) decay components correspond to nonradiative and radiative recombination, respectively. Electron-only devices (FTO/SnO_2_/perovskites/PCBM/Ag) were fabricated to calculate trap state density of the devices. The defect density was determined by the equation for the trap-filled limit voltage.

$${V}_{TFL}=\frac{\text{e}{N}_{t}{L}^{2}}{2\upvarepsilon {\upvarepsilon }_{0}}$$

The *ε*, *ε*_*0*_ and *L* are corresponded to the dielectric constant of FAPbI_3_, vacuum dielectric constant and the thickness of the perovskite film. Electrical impedance spectroscopy (EIS) was performed by using the electrochemical workstation (CHI660C, Chen Hua, China) with a frequency range from 1 Hz to 0.1 MHz in the dark. The charge transport dynamics of PSCs were analyzed using a Zahner Zennium electrochemical workstation and fitted by ZView software. Transient photovoltage decay and transient photocurrent measurements were done on a homemade system. QFLS values using the equation: QFLS = *k*_*B*_* T* * *ln* (*J*_rad_ / *J*_0, rad_) = *k*_*B*_* T* * *ln* (PLQY * *J*_gen_ / *J*_0, rad_), where *J*_rad_ is the measured total radiative equivalent current density, *J*_gen_ is the measured photogeneration current density, *J*_0,rad_ is the dark radiative recombination current density determined from the integral of the external quantum efficiency and the black body spectrum, *k*_*B*_ is Boltzmann’s constant, and *T* is the temperature. PLQY and QFLS diagram from *J–V* results for perovskite film, SnO_2_/perovskite half stack, and full cell with and without CzBP treatment. Transient photovoltage (TPV) measurements were taken on a homemade system.

#### Device Testing

*J−V* curves of the as-fabricated PSCs with different scanning directions were measured using a 2400 Source meter (Keithley, USA) under simulated 1-sun AM 1.5G 100 mW cm^−2^ intensity (Oriel Sol3A Class AAA, Newport, USA). The typical active area of PSCs is 0.090 cm^2^ defined by a metal mask from the electrode of 0.1225 cm^2^. The intensity of the 1-sun AM 1.5G illumination was calibrated using a Si-reference cell certified by the National Renewable Energy Laboratory. The scan rate during measurement was set to 200 mV s^−1^ (the voltage step is 20 mV with no delay time) from reverse and forward two scanning directions in air condition around 25 °C (RH≈25%). The External Quantum Efficiency (EQE) measurement was calculated using certified incident photon to current conversion efficiency equipment from Enlitech. The operational stability tests were performed using a stability setup (LC Auto-Test 24, Shenzhen Lancheng Technology Co., Ltd.). In the test, all PSCs were un-encapsulated, tested under continuous light illumination and maximum power point tracking. The light source consisted of an array of white LEDs (SLS-LED-80, Qingdao Solar Scientific Instrument High-tech Co., LTD) powered by a constant current. Sun intensities were calibrated by a calibrated Si-reference cell. During aging, the devices were masked and placed in a holder purged with continuous N_2_ flow. *J–V* curves with reverse voltage scans were recorded every 8 h during the whole operational test.

#### GIWAXS Simulation

GIWAXS simulation was performed using GIWAXS-Tools [12] GIWAXS patterns with preferred out-of-plane (OOP) (001) (parameters: Gaussian orientational distribution, FWHM 25°; pseudo-Voigt radical distribution, FWHM 0.02 Å^−1^), OOP (011) (parameters: Gaussian orientational distribution, FWHM 25°; pseudo-Voigt radical distribution, FWHM 0.02 Å^−1^), random, SnO_2_ (parameters: Gaussian orientational distribution, FWHM 25°; pseudo-Voigt radical distribution, FWHM 0.02 Å^−1^) and PbI_2_ (001) (Gaussian orientational distribution, FWHM 15°; pseudo-Voigt radical distribution, FWHM 0.02 Å^−1^) were simulated and merged. The cif file of (FA_0.95_Cs_0.05_PbI_3_)_0.975_(MAPbBr_3_)_0.025_ was collected by combination of cubic FAPbI_3_ [13] and MAPbBr_3_ [14]. The cell parameters were obtained from the experimental XRD results. Pole figures (I vs. χ curve) were collected in the range of *q* = 0.97–1.04 Å^−1^ and *χ* =  − 90° to 90°. For χ > 80° and χ <  − 80°, the data were not integrated because of the reflection effect. According to the scattering geometry, GIWAXS data cannot obtain signals out of the Ewald sphere (also known as the missing wedge in the *q*_z_–*q*_xy_ pattern). Under our experimental conditions (*λ* = 1.24 Å, incident angle = 0.5°), the area in the range of − 6.8° < *χ* < 6.8° cannot be obtained for *q* at 1 Å^−1^. Likewise, at an incident angle of 1.0°, − 5.0° < *χ* < 5.0° cannot be collected. The missing data were fitted using the Gaussian function and plotted in dotted curves.

#### Theoretical Calculation

Geometry optimization and frequency analysis were performed at the B3LYP-D3(BJ)/def2-SVP level using ORCA. The interaction energies were calculated using the equation: *E*_int = *E*_AB − *E*_A − *E*_B + *E*_BSSE, where *E*_AB, *E*_A and *E*_B are thermo-corrected energies of the adduct and two components; *E*_BSSE is the basis set superposition error correction term. The independent gradient model based on Hirshfeld partition (IGMH) analysis, molecular surface analysis and electrostatic potential minima was conducted by Multiwfn and visualized by VMD. The space group of the model for FAPbI_3_ is P4/NBM, and four orientations of the FA can partially mitigate the stresses associated with a single orientation. Molecular dynamics was performed by xtb [15] at the GFN1-xTB level. Data were postprocessed by Molclus [16]. All conformations were aligned and visualized by VMD.

First-principles calculations were performed by Vienna Ab initio Simulation Package (VASP) [17] with a cutoff of 400 eV. A generalized gradient approximation is in the form of PBEsol functionals to simulate the exchange–correlation interactions. Coordination of CzBP molecules in the bulk was calculated by a dual-interface model. Coordination of CzBP at the surface was conducted by constructing a slab and a vacuum layer of 15 Å. The optimized structures and charge density differences were visualized by VESTA [18].

## Results and Discussion

### Theoretical Study of 2,7-CzBP and 3,6-CzBP

We accessed both 2,7- and 3,6-CzBP via a Pd-catalyzed cross-coupling in 80% yields (Figs. S1-S4). CzBP molecules exhibit good solubility and conductivity (Fig. S5). Theoretical calculation suggests that 2,7-CzBP has more extended conjugation than 3,6-CzBP, as evidenced by the reduced highest occupied molecular orbital (HOMO)–lowest unoccupied molecular orbital (LUMO) gap (Fig. S6) and ultraviolet–visible (UV) spectra (Fig. S7) [19]. These bisphosphonate molecules have a conformational preference with a O = P···carbazole dihedral angle of smaller than 10° at the energetic minimum. However, the rotational barriers for the phosphonate groups are small (< 2 kcal mol^−1^), indicating the coexistence of different conformations (Figs. S8-S10). Electrostatic potential (ESP) isosurfaces of the CzBP molecules show that the surface minimum is localized at P = O of the phosphonate and the maximum at –NH of carbazole (Figs. 1b and S11). Different conformations have similar energies and ESP-related molecular descriptors (i.e., restrained ESP charges and molecular polarity index [20], Fig. S12). The rotational flexibility of phosphonate groups is further confirmed by molecular dynamics (MD) calculations (Fig. 1c). MD trajectories indicate that the average angles between two phosphonate groups are 147° and 93° for 2,7-CzBP and 3,6-CzBP, respectively. Binding energies show that the two molecules preferred different passivation modes (Fig. 1d). 2,7-CzBP has a near-linear molecular geometry that increases the possibility to parallelly pack and fill the gap. This feature may help to bridge the crystallites and improve charge transport. By contrast, 3.6-CzBP has a V-shaped geometry that dominantly interacts with the one-sided perovskite surface. Such isomerism effect suggests that the two carbazolyl bisphosphonate molecules are both good passivators but have different influences on charge transport properties (Fig. 1e).

### Characterization of 2,7- and 3,6-CzBP-Treated Perovskite Films

**Fig. 2:**
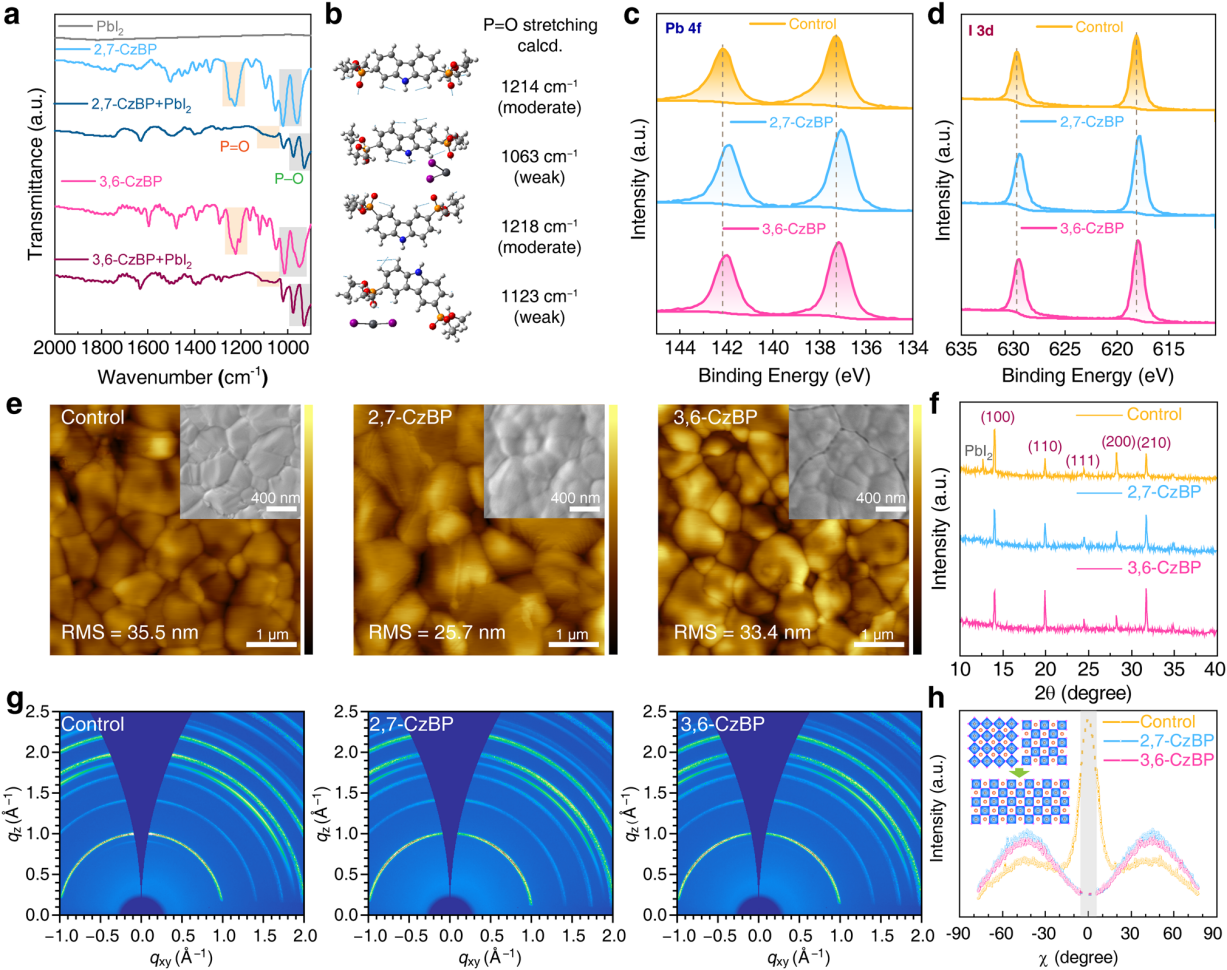
Interactions between CzBP and perovskites and films morphology. **a** FTIR spectra of CzBPs and their mixture with PbI_2_. **b** IR vibrations calculated at the B3LYP-D3(BJ)/def2-SVP level (scale factor: 0.96). XPS curves of **c** Pb *4f*, **d** I *3d* binding energies of perovskite films. **e** AFM images and top-view SEM images (inset) of perovskite films. **f** XRD patterns, **g** GIWAXS patterns of the control and the CzBP-treated perovskites. **h** Pole figure of (100) diffraction of perovskite films (inset: schematics of the orientation preferences after CzBP addition)

To unveil interactions between CzBP and perovskite, we conducted FTIR spectroscopy and XPS. Peaks at ca. 1230 cm^−1^ in the FTIR spectra of both CzBP molecules were attributed to P = O stretching (Fig. 2a). Interestingly, after addition of PbI_2_, these peaks visually disappeared. Theoretical analyses suggest a substantial downshift (almost 100 cm^−1^) and reduced strength of the P = O stretching peak upon coordination with Pb^2+^ (Fig. 2b), consistent with the IR spectra. Therefore, such phenomena signified a coordination event through P = O···Pb interactions. N–H asymmetrical stretching showed a similar tendency (Fig. S13), demonstrating the presence of P = O···H–N interactions. Moreover, XPS peaks for Pb *4f* and I *3d* shifted to lower binding energies, confirming the effective anchoring of CzBP to perovskites (Fig. 2c, d). Such interaction creates a molecular lock within the perovskite structure, allowing for enhanced relaxation time for ion migration [21, 22].

We then explored the morphology and microstructure of perovskite films. The AFM images unveiled that CzBP-treated films exhibited a uniform and dense surface (Fig. 2e). Top-view SEM images corroborated this observation, revealing a smoother surface and a larger grain size in the 2,7-CzBP-treated film. XRD patterns substantiated the increased grain sizes with narrowed full widths at half maximum by addition of CzBP (Figs. 2f and S14) [23, 24]. All patterns displayed characteristic cubic *α*-phase peaks, indicating CzBP accumulation at grain boundaries rather than lattice insertion [25, 26]. Notably, the control film depicted a PbI_2_ peak at 12.68°, which was absent in CzBP-treated films because of the coordination. The grazing-incidence wide-angle X-ray scattering (GIWAXS) images (Fig. 2g) agree with the XRD patterns. The *I*–*χ* pole figure integrated at *q* = 0.97–1.04 Å^−1^ exhibited a bimodal packing with peaks at both *χ* = 0° and 45° for the control film. By contrast, the CzBP-treated films exhibited a preferred out-of-plane (110) orientation with a single peak at 45° (Fig. 2h). Similar GIWAXS patterns were observed at a larger incident angle of 1.0° with a penetration depth of 295 nm (Figs. S15–S17). All these scattering patterns were well-matched with the GIWAXS simulation (Fig. S18). These results elucidate the impact of CzBP treatment on perovskite crystalline orientation. The CzBP additive reduces the surface energy and changes the growth rates of specific crystal planes, resulting in highly ordered polycrystalline films with oriented growth [27, 28]. This reduced surface energy contributes to the equilibrium shape, as predicted by Wulff construction [29]. These findings provide a robust foundation for improved film uniformity and growth orientation.

**Fig. 3:**
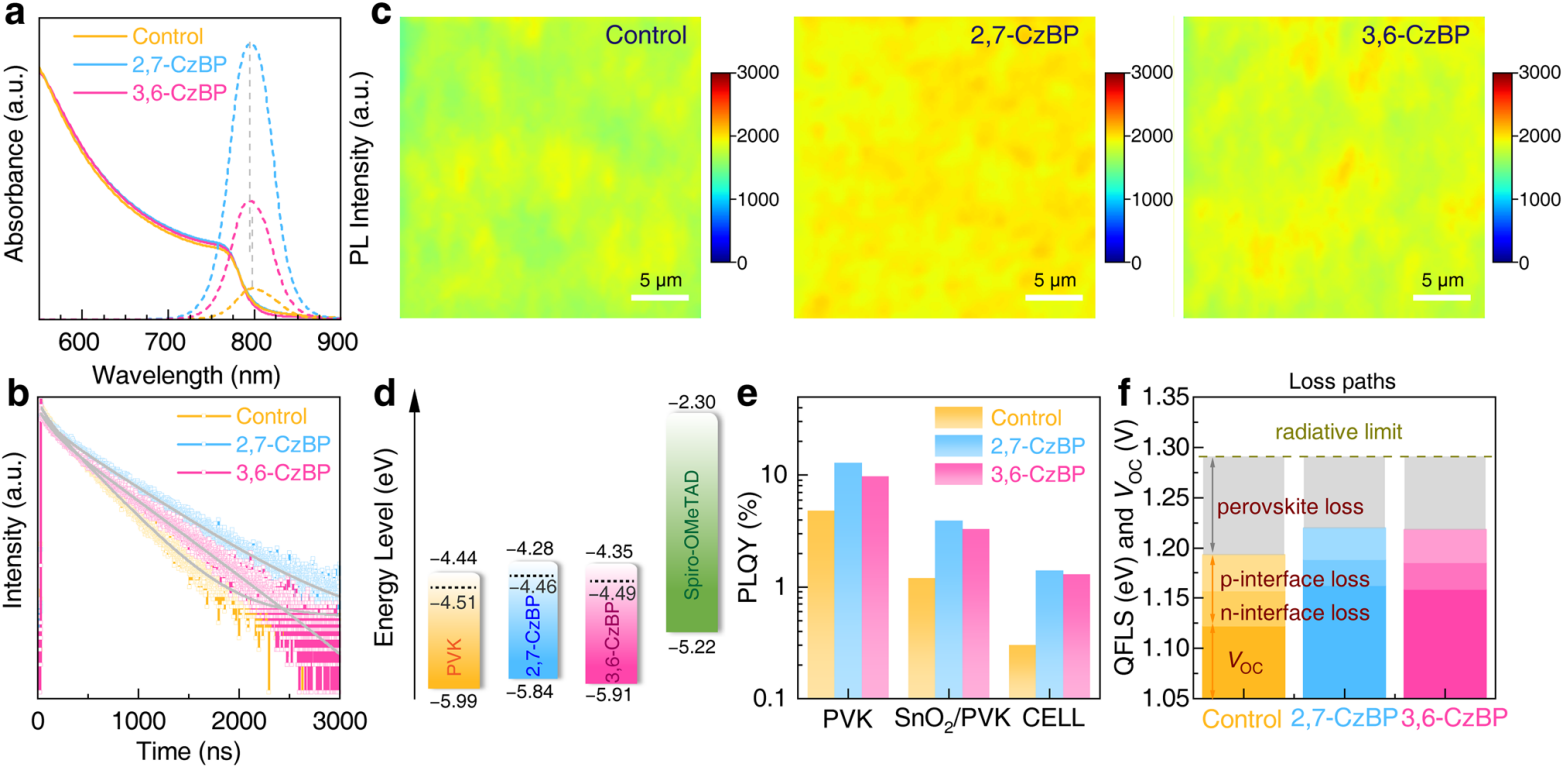
Characterization of perovskite quality. **a** UV–Vis absorption and steady-state PL spectra. **b** Time-resolved PL spectra of perovskite films. **c** PL mapping of perovskite films. **d** Energy level of perovskite layers in PSC (dashed black lines: Fermi levels). **e** PLQY values of different structures, **f** internal QFLS diagram

We then characterized the optoelectronic properties of perovskite films to understand how the molecules affect device performance. The UV–Vis absorption spectra showed similar absorption with an optical gap (*E*_g_) of 1.56 eV for all samples (Figs. 3a and S19). However, the steady-state photoluminescence (PL) intensity was substantially enhanced after CzBP treatment (Fig. 3a). This observation is correlated with suppressed nonradiative recombination [30, 31]. Time-resolved photoluminescence (TRPL) measurement exhibited extended carrier lifetimes, from 348.15 ns for the bare perovskite to 481.16 ns for 3,6-CzBP and 560.59 ns for 2,7-CzBP-treated films (Fig. 3b and Table S1). The extended carrier lifetime reflects reduced nonradiative traps and enhanced photon recirculation within the perovskite films [32, 33]. Moreover, PL mapping shows an overall higher and more uniform PL emission with CzBP treatment (Fig. 3c), in line with the longer PL lifetimes. This feature potentially confers advantages to fabrication of large-area PSCs.

From ultraviolet photoelectron spectroscopy, we show that CzBP treatment led to favorable band alignment with the hole transport material (HTM) and thus reduced the hole transport barrier (Figs. 3d and S20) [34, 35]. Notably, the 2,7-CzBP treatment led to a more energetically favorable alignment compared with the 3,6-CzBP treatment. This improved alignment promotes efficient hole extraction and reduces nonradiative carrier recombination, ultimately leading to better device performance [36]. Photoluminescence quantum yield (PLQY) measurement on partial cell stacks and subsequent estimation of quasi-Fermi-level splitting (QFLS) at the interface allowed for further quantification of nonradiative recombination losses (Fig. 3e, f). Compared with 4.8% for bare perovskite, both 2,7- and 3,6-CzBP samples exhibited higher PLQY values of 12.9% and 9.7%, respectively. The PLQY value of electron transport material (ETM)/PVK and ETM/PVK/HTM on glass showed the same trend, indicating that treatment of CzBP minimizes nonradiative recombination loss by improving energy-level alignment and passivating defects. The ETM/PVK half stack exhibited a lower QFLS of 1.158 eV, while CzBP treatment led to higher QFLS values (1.189 eV for 2,7-CzBP and 1.186 eV for 3,6-CzBP) with the ETM. These values are comparable to the bare perovskite, suggesting effective suppression of recombination [37, 38]. With a radiative limit of 1.29 eV for the given bandgap, the voltage loss of the device is dominated by perovskite and interface loss. Treatment with CzBP effectively suppresses these pathways, contributing to a higher *V*_OC_.

### Photovoltaic Performance and Stability of PSCs

**Fig. 4:**
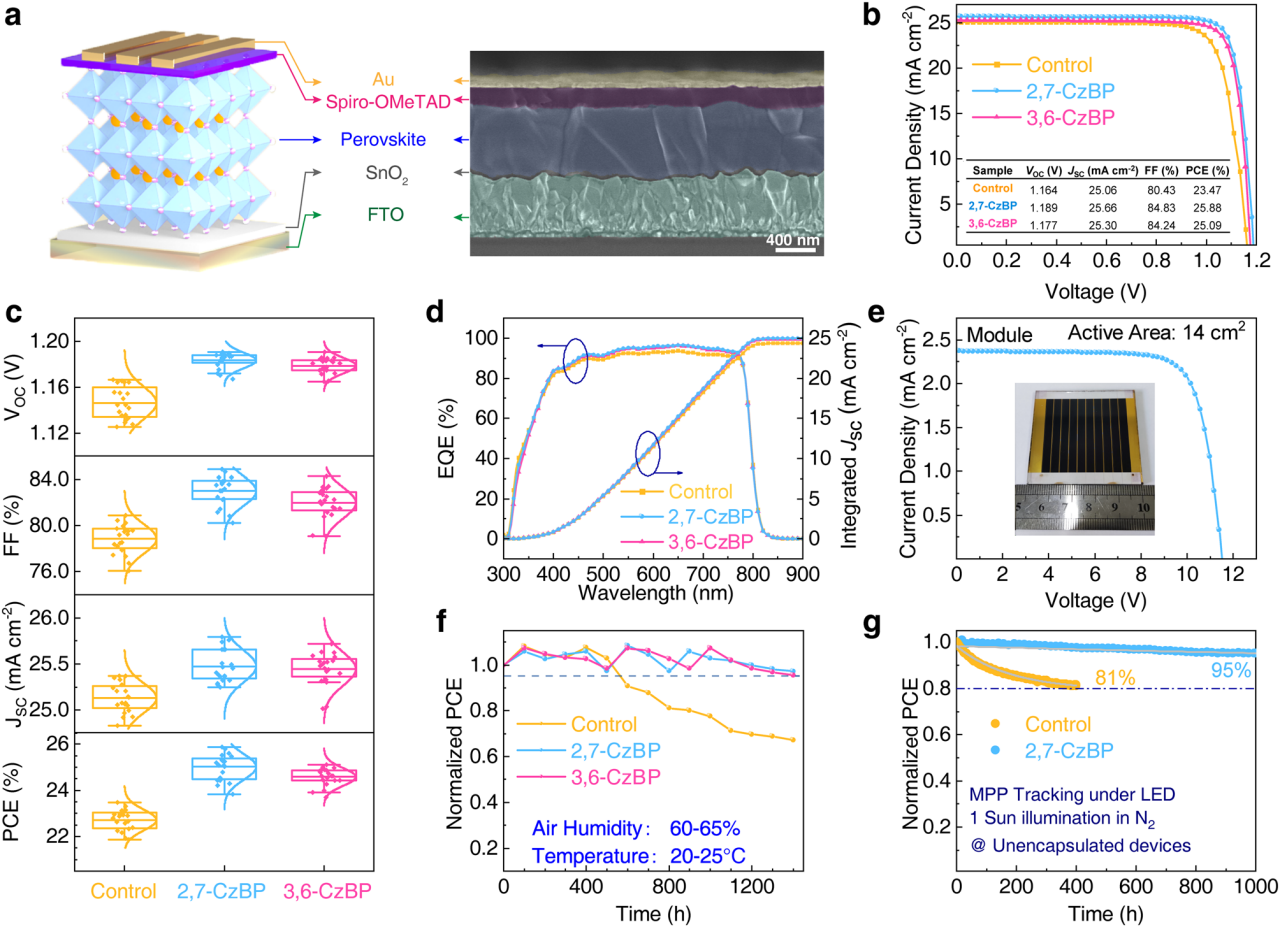
Device performance and characteristic of the PSCs. **a** Device structure and cross-sectional SEM image. **b** *J–V* curves of champion PSCs. **c** Performance statistics of *V*_OC_, FF, *J*_SC_ and PCE. **d** EQE and integrated *J*_SC_ curves. **e** *J–V* curves of a 14 cm^2^ module with 2,7-CzBP treatment. **f** Stability of the devices in high ambient humidity without encapsulation. **g** MPP tracking in nitrogen at 25 °C

We fabricated PSCs with a device structure of fluorine-doped tin oxide (FTO)/SnO_2_/PVK/spiro-OMeTAD/Au (Fig. 4a). The optimal concentrations of the precursor solution were 0.5 mg mL^−1^ for 2,7-CzBP and 0.3 mg mL^−1^ for 3,6-CzBP (Fig. S21). The champion PCE increased from 23.47% for the control to 25.88% for the 2,7-CzBP-treated device (Fig. 4b). The champion device had a *V*_OC_ of 1.189 V, a FF of 84.83% and a *J*_SC_ of 25.66 mA cm^−2^. The 3,6-CzBP-treated device also presents an improved PCE of 25.09%. Figure 4c and Table S2 list the statistical data of PSCs. The reverse and forward *J–V* scans displayed hysteresis of 5.5%, 3.2% and 3.9% for the control, 2,7-CzBP and 3,6-CzBP devices, respectively (Fig. S22 and Table S3). The external quantum efficiency (EQE) spectra and integrated *J*_SC_ are also recorded (Fig. 4d). To evaluate photostability and reproducibility, we conducted steady-state output (SPO) measurement for 450 s (Fig. S23) and prepared 60 devices for the histogram of their PCE values based on each optimal condition (Fig. S24). Encouraged by the promising performance of the CzBP-treated devices, we sought to explore their potential for large-area applications. We fabricated PSCs modules using 2,7-CzBP (Fig. 4e). The champion 2,7-CzBP-treated 14 cm^2^ module achieved a PCE of 21.04% with a *V*_OC_ of 11.53 V, a *J*_SC_ of 2.37 mA cm^−2^ and a FF of 76.86%. These results demonstrate the high potential of CzBP analogs for large-area PSC fabrication.

To assess the impact of CzBP on device stability under environmental factors, we systematically compared the CzBP-treated and untreated devices under controlled humidity and temperature conditions. Aging in the air with 60%–65% relative humidity (RH) and 20–25 °C temperature (Fig. 4f), the unencapsulated CzBP-treated devices displayed remarkable resilience. While the control device degraded by > 30% after 1400 h, unencapsulated CzBP-treated devices retained > 95% of their initial efficiency (97.02% for 2,7-CzBP and 95.48% for 3,6-CzBP). The enhanced stability is primarily due to the improved hydrophobicity with CzBP treatment. Larger water contact angles for CzBP-treated films (78.7° for 2,7-CzBP and 76.0° for 3,6-CzBP) than the bare perovskite (60.5°) indicate greater resistance against moisture intrusion (Fig. S25). CzBP treatment also led to improved thermal stability, as demonstrated by heating experiments at 65 °C (Figs. S26 and S27). The 2,7-CzBP-treated device retained 85.96% of the initial PCE after 1400 h of heating in air and 77.07% after 400 h in nitrogen, much better than the control devices (55.93% and 24.32%). The maximum power point (MPP) tracking at 1-sun illumination with a white-light-emitting diode (LED) test confirms the stability advantage of CzBP-treated devices (Fig. 4g). While the control device retained only 81% of its initial efficiency after 400 h, the unencapsulated 2,7-CzBP-treated device impressively maintained 95% after 1000 h. Our observation corroborates the enhanced device stability under thermal and humidity stress. This improvement is attributed to the multi-site coordination between CzBP and the perovskite, allowing strengthened anchoring of ions and reduced defect densities for high-quality films.

### Charge-Carrier Dynamics of 2,7- and 3,6-CzBP-Treated PSCs

**Fig. 5:**
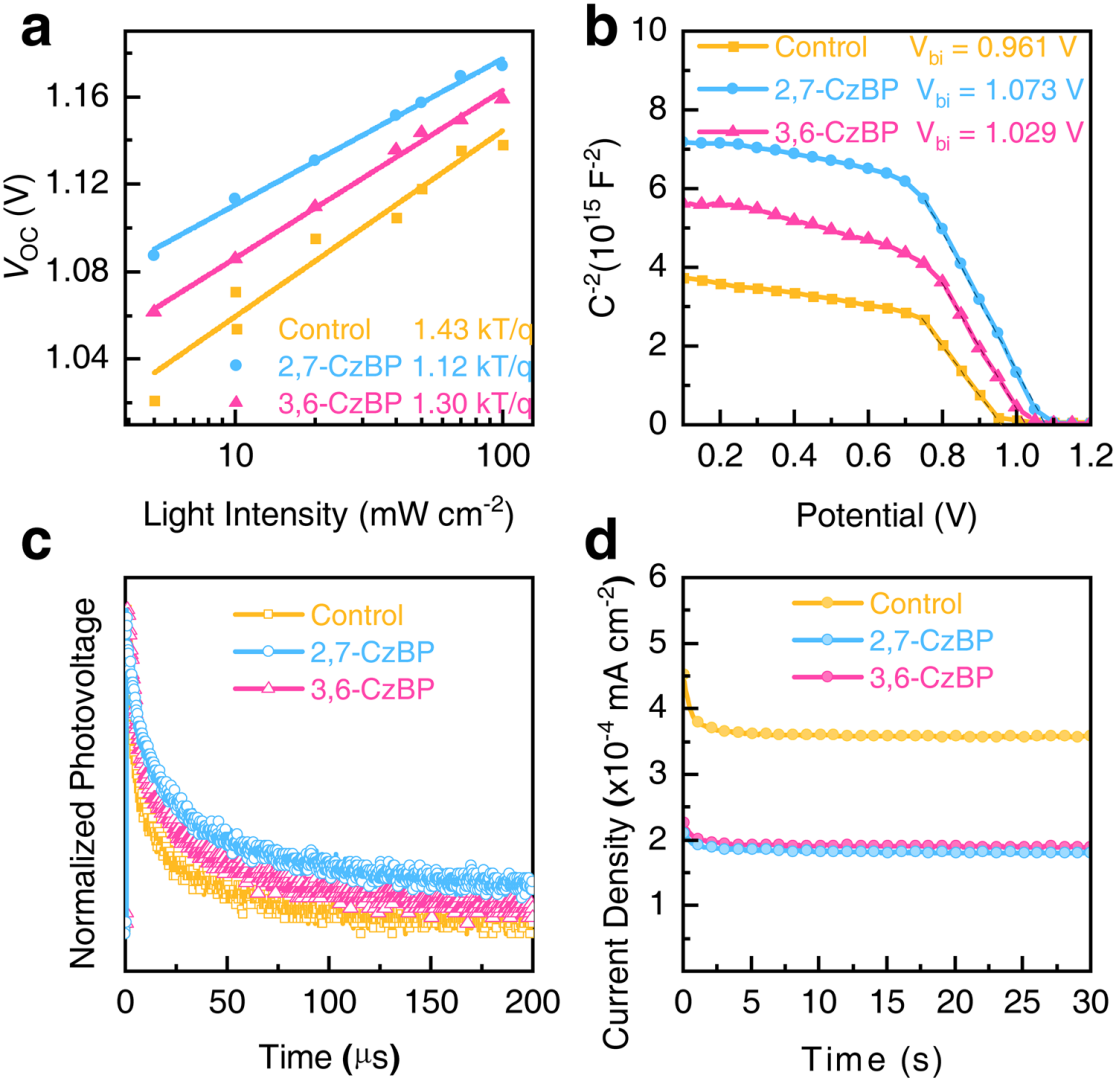
Passivation effect and ion migration of the PSCs. **a** Dependence of *V*_OC_ on light intensity. **b** Mott−Schottky plots, **c** TPV at the open circuits. **d** Transient ion currents for extracting mobile ion concentrations

We then investigated the trap-induced defect density profiles to understand the passivation effect of CzBP for the cells through the space charge limit current (SCLC) method, light intensity-dependent *V*_OC_ and *J*_SC_ and dark *I–V* measurement. The 2,7-CzBP-treated devices showed the lowest *N*_t_ of 6.97 × 10^15^ cm^−3^ (Fig. S28). Furthermore, the reduced slope of the light intensity-dependent *V*_OC_ in *n* value indicates suppressed Shockley–Read–Hall recombination in the CzBP-treated devices (Fig. 5a) [39, 40]. Light intensity-dependent *J*_SC_ plots (Fig. S29) show α values closer to 1 for CzBP-treated devices. Notably, CzBP-treated devices demonstrated a lower leakage current than the control (Fig. S30), further confirming the reduced interfacial nonradiative recombination.

To quantitatively analyze charge transport and recombination kinetics, we performed electrochemical impedance spectroscopy (EIS), Mott–Schottky plot, TRPL, transient photovoltage (TPV) and transient photocurrent (TPC) measurements. CzBP-treated devices exhibited larger semicircle fits in EIS plots, indicating reduced interfacial carrier recombination rate (Fig. S31 and Table S4). Moreover, the Mott–Schottky analysis revealed an improved built-in potential (*V*_bi_) of 1.073 V for the 2,7-CzBP-treated device compared with 0.961 V for the control device (Fig. 5b). This improvement internally drives carrier separation and transport, contributing to the high *V*_OC_’s in the CzBP-treated devices [41]. We fabricated devices with HTM on the perovskite film for TRPL measurements to further evaluate charge recombination kinetics at the PVK/HTM interface (Fig. S32 and Table S5). CzBP-treated perovskite films showed sharply decreased emission for more efficient hole extraction [42]. This improved carrier separation contributes to a higher overall device efficiency by minimizing energy losses from unwanted recombination processes.

TPV and TPC measurements were taken under 1-sun white LED illumination (Figs. 5c and S33). The 2,7-CzBP-treated device exhibited a slowed down photovoltage decay lifetime of 51.06 *μ*s compared with 30.74 *μ*s for the control ones. This result corroborates the PLQY results, both indicative of reduced trap-assisted nonradiative recombination. Furthermore, the carrier transient time from TPC measurements was 2.61 *μ*s for 2,7-CzBP-treated devices, shorter than that of the control device (4.73 *μ*s). This suggests an enhanced vertical carrier extraction efficiency to suppress hysteresis [43]. The transient ion-diffusion currents (Fig. 5d) under dark conditions illustrate that the CzBP-treated devices had lower currents and shorter decay times after applying an external forward bias at *V*_OC_ of 1.5 V for 1 min. Additionally, the 2,7-CzBP and 3,6-CzBP-treated devices exhibited lower mobile ion concentrations of 8.55 × 10^16^ and 8.97 × 10^16^ cm^−3^, respectively, compared with 1.79 × 10^17^ cm^−3^ in the control devices. The mobile ion charging–discharging results suggest that the CzBP additives suppress ion migration, thus contributing to improved device performance and long-term stability [44, 45].

## Conclusions

In summary, we study the isomerism effect of carbazolyl bisphosphonate additives. The bisphosphonate units have strong capabilities to passivate defects and suppress degradation. The different substitution patterns, however, lead to distinct dual-passivation modes. The bisphosphonate substituted at 2,7-positions of carbazole allows more effective conjugation and a near-linear molecular shape that facilitate charge transport, resembling single-molecule junctions. 2,7-CzBP-treated devices achieved 25.88% and 21.04% efficiencies for areas of 0.09 and 14 cm^2^, respectively. Our work may inspire new design strategies for conjugated organic additives for high efficiency, stable PSCs.

## Supplementary Information

Below is the link to the electronic supplementary material.

Supplementary file1 (DOCX 6912 KB)
