# Supplementary material for: Tuning Isomerism Effect in Organic Bulk Additives Enables Efficient and Stable Perovskite Solar Cells
Source: Nanomicro Lett. 2025 Jan 10;17:107. doi: 10.1007/s40820-024-01613-z (PMC11723880; doi:10.1007/s40820-024-01613-z)
Supplement: Supplementary file 1 — Supplementary file1 (DOCX 6912 KB) [file 40820_2024_1613_MOESM1_ESM.docx]

Supporting Information for

**Tuning Isomerism Effect in Organic Bulk Additives Enables Efficient and Stable Perovskite Solar Cells**

Qi Zhang^1^, Qiangqiang Zhao^1^, Han Wang^2^, Yiguo Yao^1^, Lei Li^1^, Yulin Wei^1^, Ruida Xu^1^, Chenyang Zhang^1^, Erik O. Shalenov^4^, Yongguang Tu^1,^*, Kai Wang^1,^ * and Mingjia Xiao^3,^*

^1^Institute of Flexible Electronics (IFE), Northwestern Polytechnical University (NPU), Xi’an 710072, P. R. China

^2^School of Management, Xián Polytechnic University, Xi’an 710072, P. R. China

^3^The Quzhou Affiliated Hospital of Wenzhou Medical University, Quzhou People’s Hospital, Quzhou 324000, P. R. China

^4^ Institute of Experimental and Theoretical Physics, al-Farabi Kazakh National University, Almaty 050040, Kazakhstan

*Corresponding authors. E-mail: [kaiwang@nwpu.edu.cn](mailto:kaiwang@nwpu.edu.cn) (Kai Wang); [iamygtu@nwpu.edu.cn](mailto:iamygtu@nwpu.edu.cn) (Yongguang Tu); [qyctmb@wmu.edu.cn](mailto:qyctmb@wmu.edu.cn) (Mingjia Xiao)

**Supplementary Figures and Tables**

 **Scheme S1** 2,7-CzBP synthetic route

To a three-necked flask were added 2,7-dibromocarbazole (1.00 g, 3.08 mmol), diethyl phosphite (1.00 g, 7.24 mmol), Pd(PPh_3_)_4_ (0.356 g, 0.308 mmol), dry toluene (50 mL) under nitrogen protection. After the reactants were dissolved, triethylamine (1.3 mL) was added. The mixture was reacted overnight at 100 °C. After the mixture was allowed to cool to room temperature, the precipitate was filtered, washed with cold toluene, and concentrated under vacuum to remove the solvent and the excess diethyl phosphite. The crude product was purified by column chromatography (eluent: CH_2_Cl_2_/MeOH 50:1, v/v). The oily product was sonicated in *n*-hexane to afford a white powder (1.10 g, yield: 81.4%). ^1^H NMR (500 MHz, CDCl_3_) δ 10.60 (s, 1H), 8.28 (d, *J* = 14.9 Hz, 2H), 8.23 (dd, *J* = 8.0, 3.9 Hz, 2H), 7.65 (m, 2H), 4.30–4.11 (m, 8H), 1.37 (t, *J* = 7.0 Hz, 12H). ^13^C NMR (125 MHz, Chloroform-*d*) δ 140.21 (d, *J* = 20.7 Hz), 125.49 (d, *J* = 187.6 Hz), 125.43 (d, *J* = 2.7 Hz), 121.47 (d, *J* = 10.0 Hz), 121.13 (d, *J* = 17.0 Hz), 116.28 (d, *J* = 12.0 Hz), 62.32 (d, *J* = 5.2 Hz), 16.37 (d, *J* = 6.5 Hz). HRMS (ESI, m/z): calcd for C_20_H_28_NO_6_P_2_ [M+H]^+^, 439.3845; found, 439.3834.

 **Scheme S2** 3,6-CzBP synthetic route

The synthetic route to 3,6-CzBP was similar to 2,7-CzBP. The product was a white powder (1.08 g, yield: 79.9%). ^1^H NMR (500 MHz, CDCl_3_) δ 9.14 (s, 1H), 8.70–8.57 (m, 2H), 7.92 (m, 2H), 7.58 (m, 2H), 4.27–4.10 (m, 8H), 1.38 (t, *J* = 7.1 Hz, 12H). ^13^C NMR (125 MHz, Chloroform-*d*) δ 142.37 (d, *J* = 3.0 Hz), 129.64 (d, *J* = 12.0 Hz), 125.39 (d, *J* = 11.0 Hz), 122.79 (d, *J* = 17.5 Hz), 118.69 (d, *J* = 192.8 Hz), 111.30 (d, *J* = 16.5 Hz), 62.15 (d, *J* = 5.4 Hz), 16.40 (d, *J* = 6.6 Hz). HRMS (ESI, m/z): calcd for C_20_H_28_NO_6_P_2_ [M+H]^+^, 439.3845; found, 439.3832.

**Fig. S1** ^1^H NMR spectrum of 2,7-CzBP


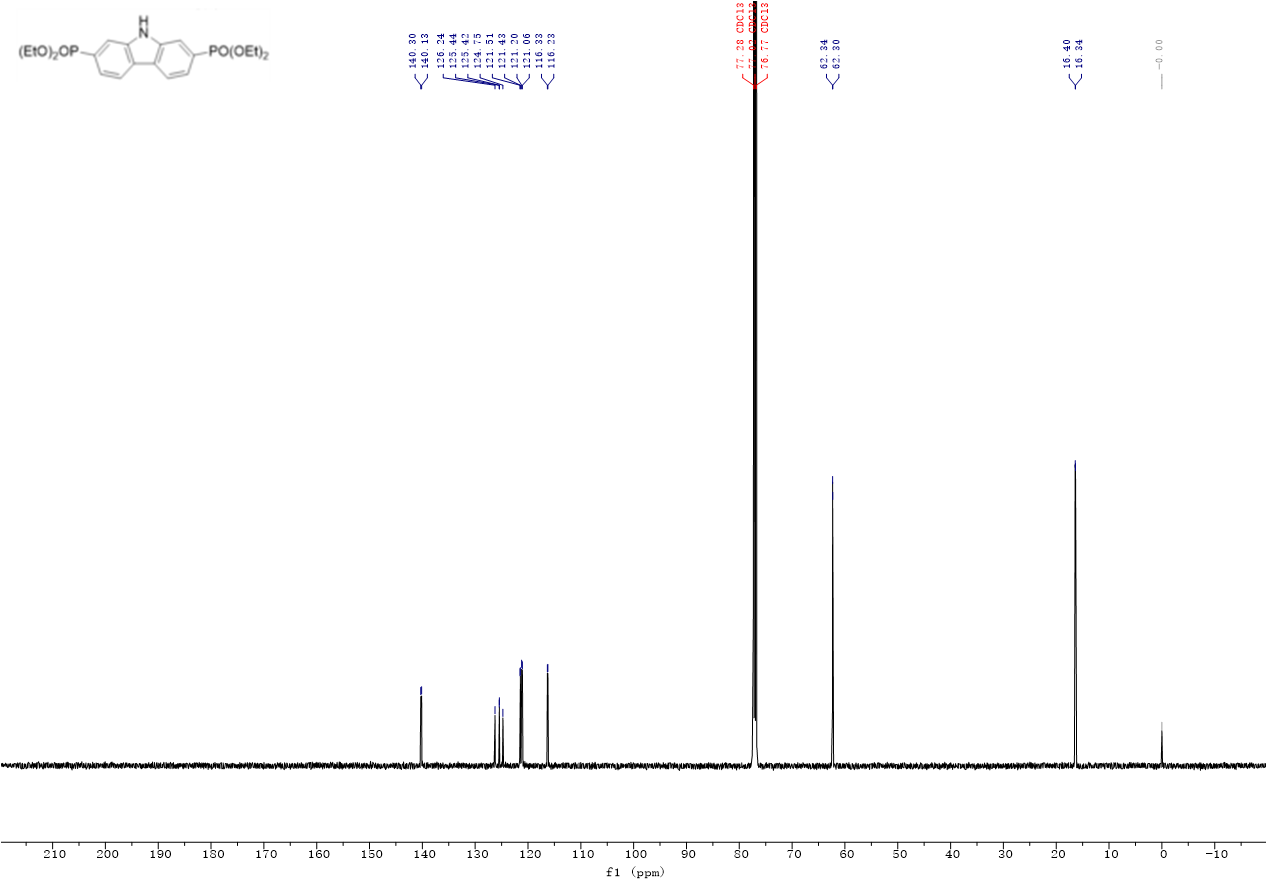


**Fig. S2** ^13^C NMR spectrum of 2,7-CzBP

**Fig. S3** ^1^H NMR spectrum of 3,6-CzBP

**Fig. S4** ^13^C NMR spectrum of 3,6-CzBP


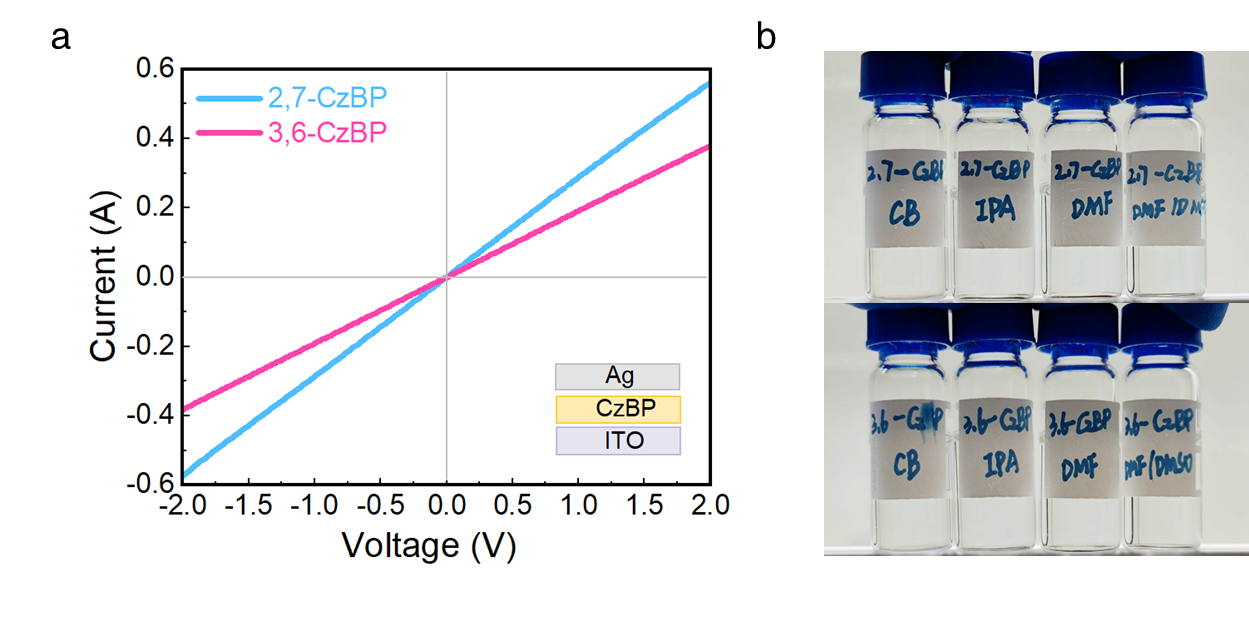


**Fig. S5** **a** Conductivity and **b** solubility of CzBP molecules


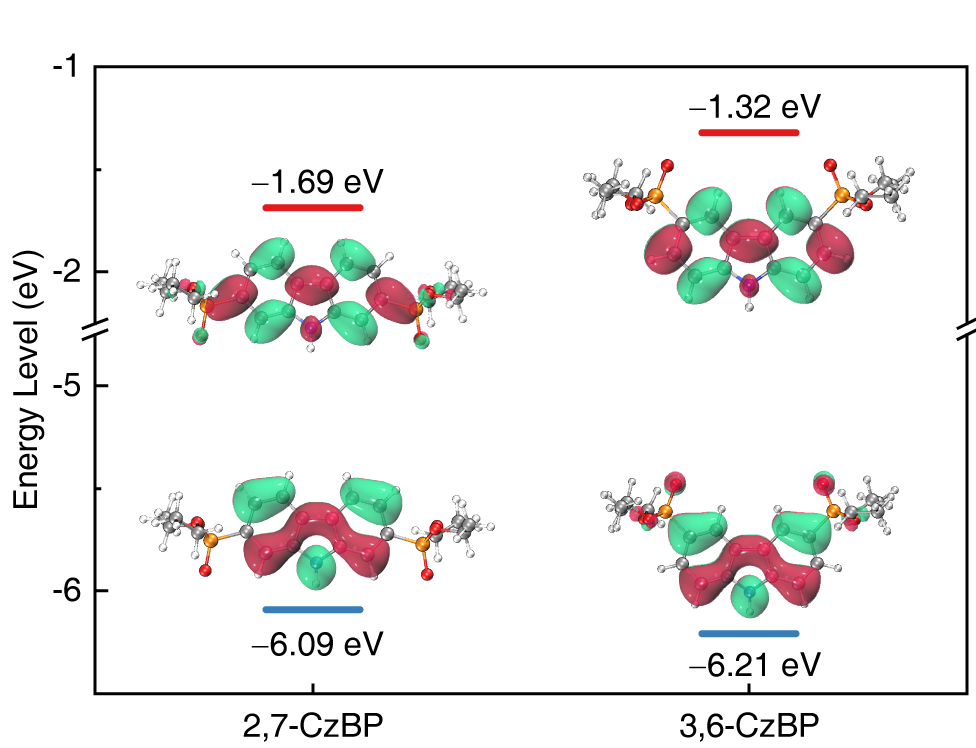


**Fig. S6** Frontier molecular orbitals of 2,7-CzBP and 3,6-CzBP calculated at the B3LYP-D3(BJ)/def2-SVP level


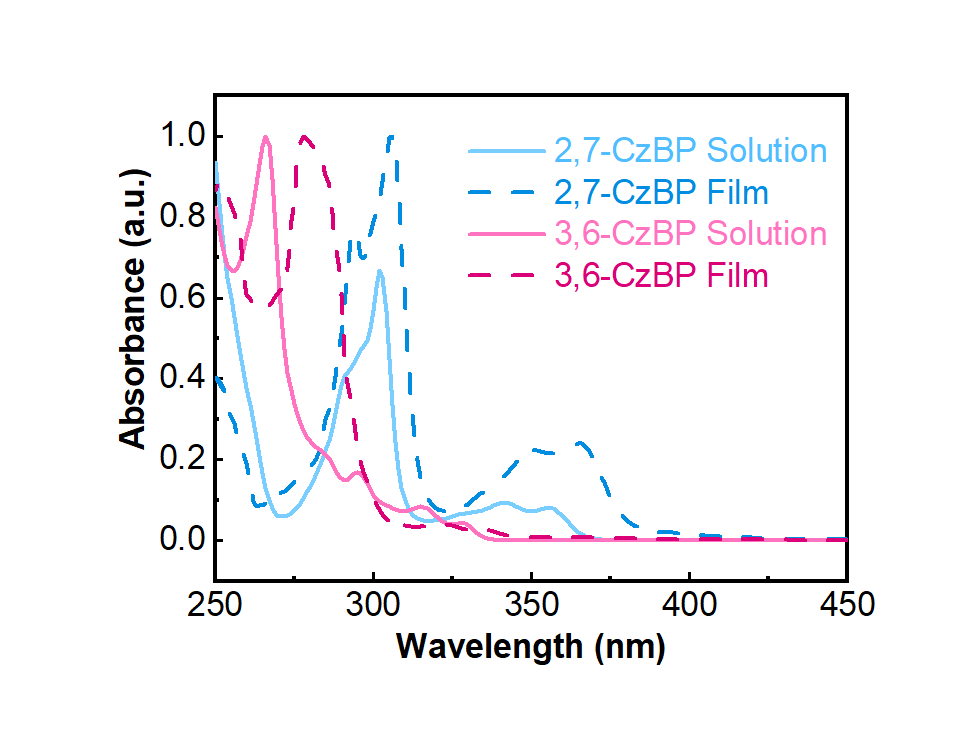


**Fig. S7** Normalized absorption spectra of 2,7-CzBP and 3,6-CzBP in their thin films and chloroform solutions at room temperature


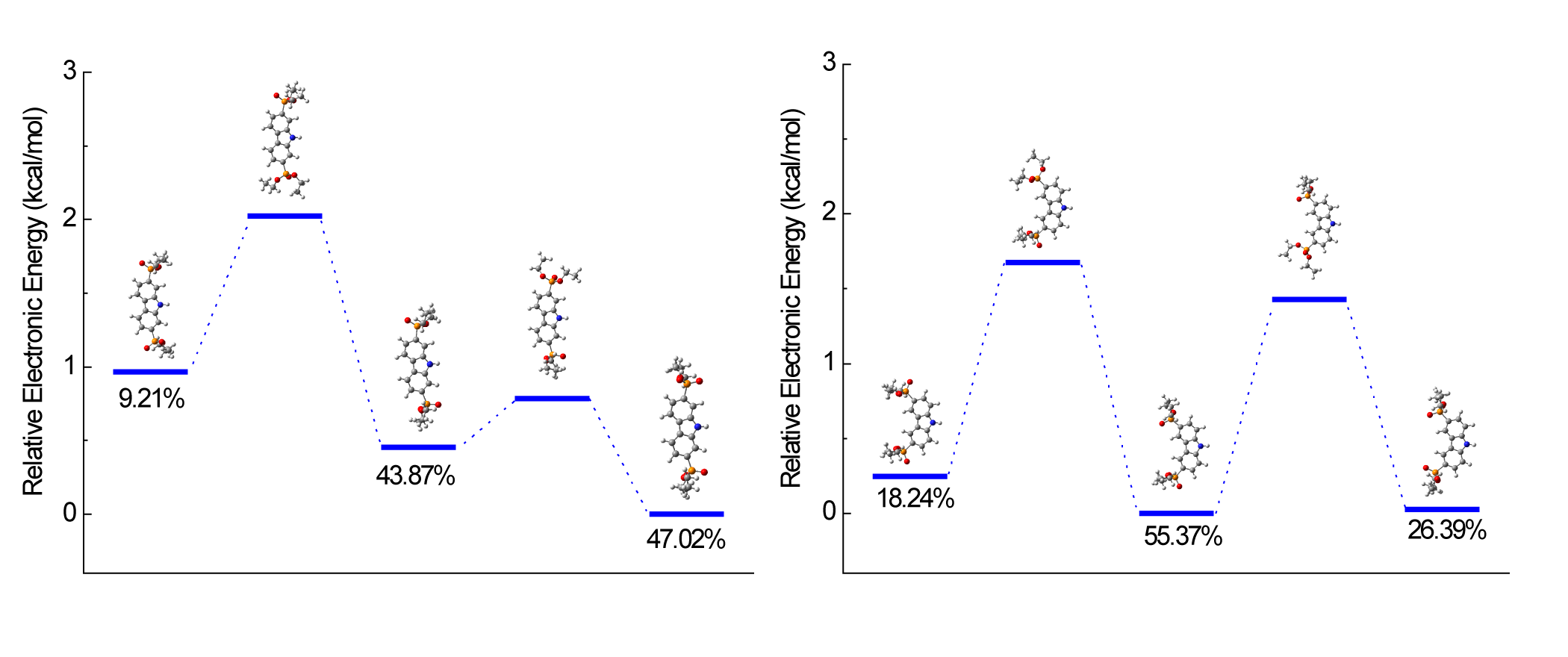
**Fig. S8** Relative electronic energies, rotational barriers and Boltzmann distributions (298.15 K) of different conformers for 2,7-CzBP and 3,6-CzBP, calculated at the B3LYP-D3(BJ)/def2-SVP level


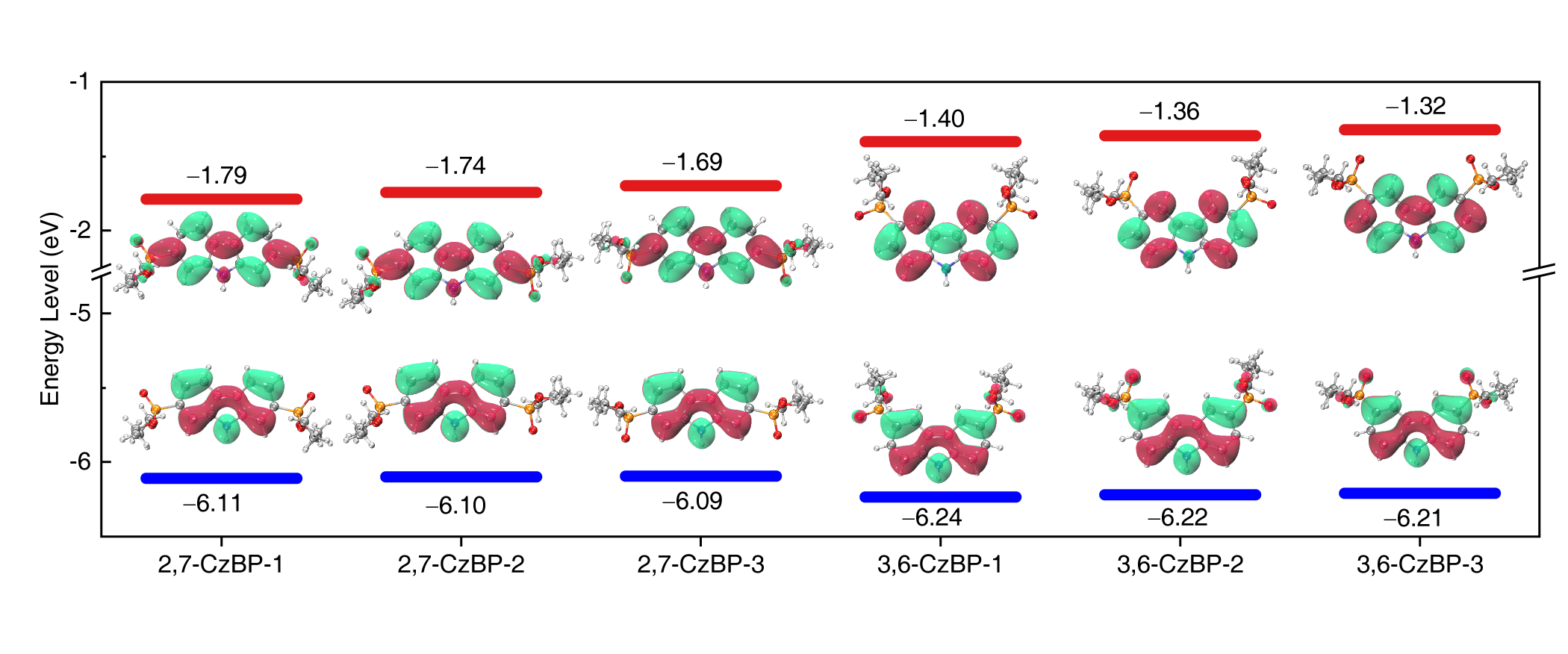


**Fig. S9** Frontier molecular orbital isosurfaces (0.02 a.u.) and energy levels of different conformers for 2,7-CzBP and 3,6-CzBP calculated at the B3LYP-D3(BJ)/def2-SVP level


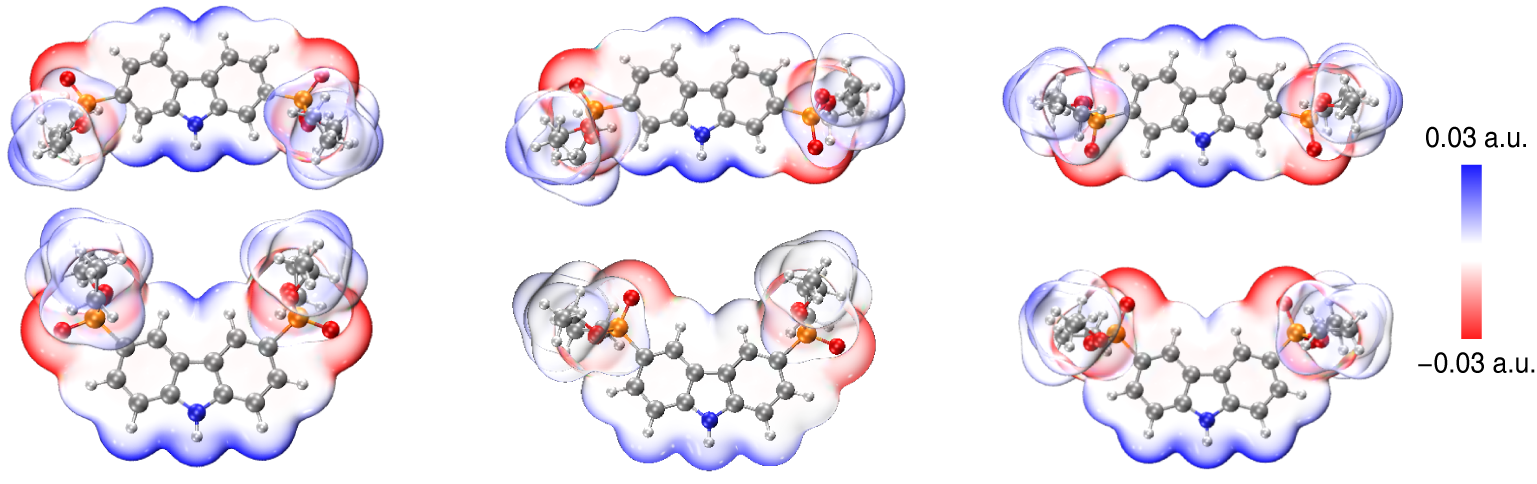


**Fig. S10** Electrostatic potential isosurfaces (isovalue: 0.001 a.u.) of different conformers for 2,7-CzBP and 3,6-CzBP calculated at the B3LYP-D3(BJ)/def2-SVP level


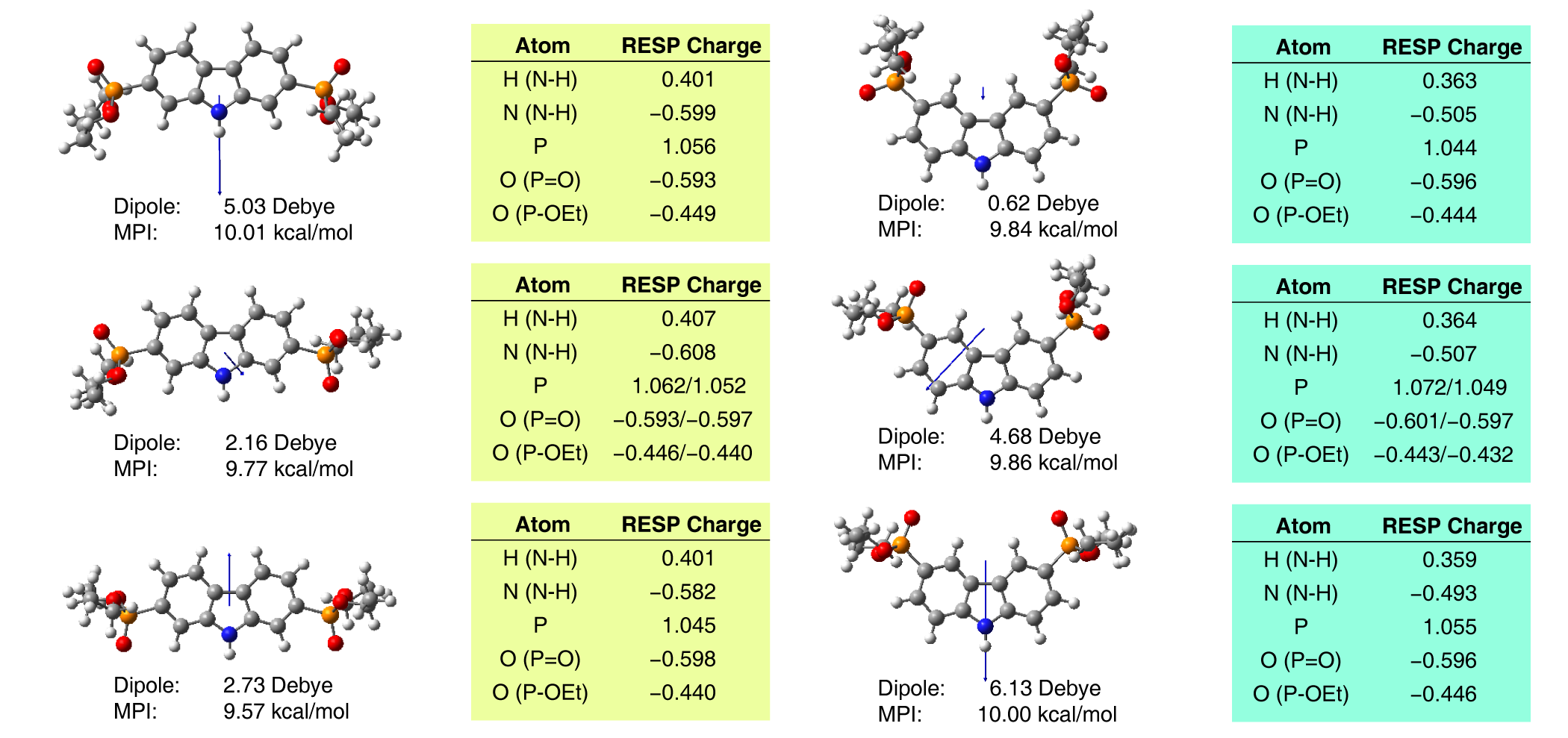


**Fig. S11** Restrained electrostatic potential charges, dipole moments, and molecular polarity indices of different conformers for 2,7-CzBP and 3,6-CzBP calculated at the B3LYP-D3(BJ)/def2-SVP level


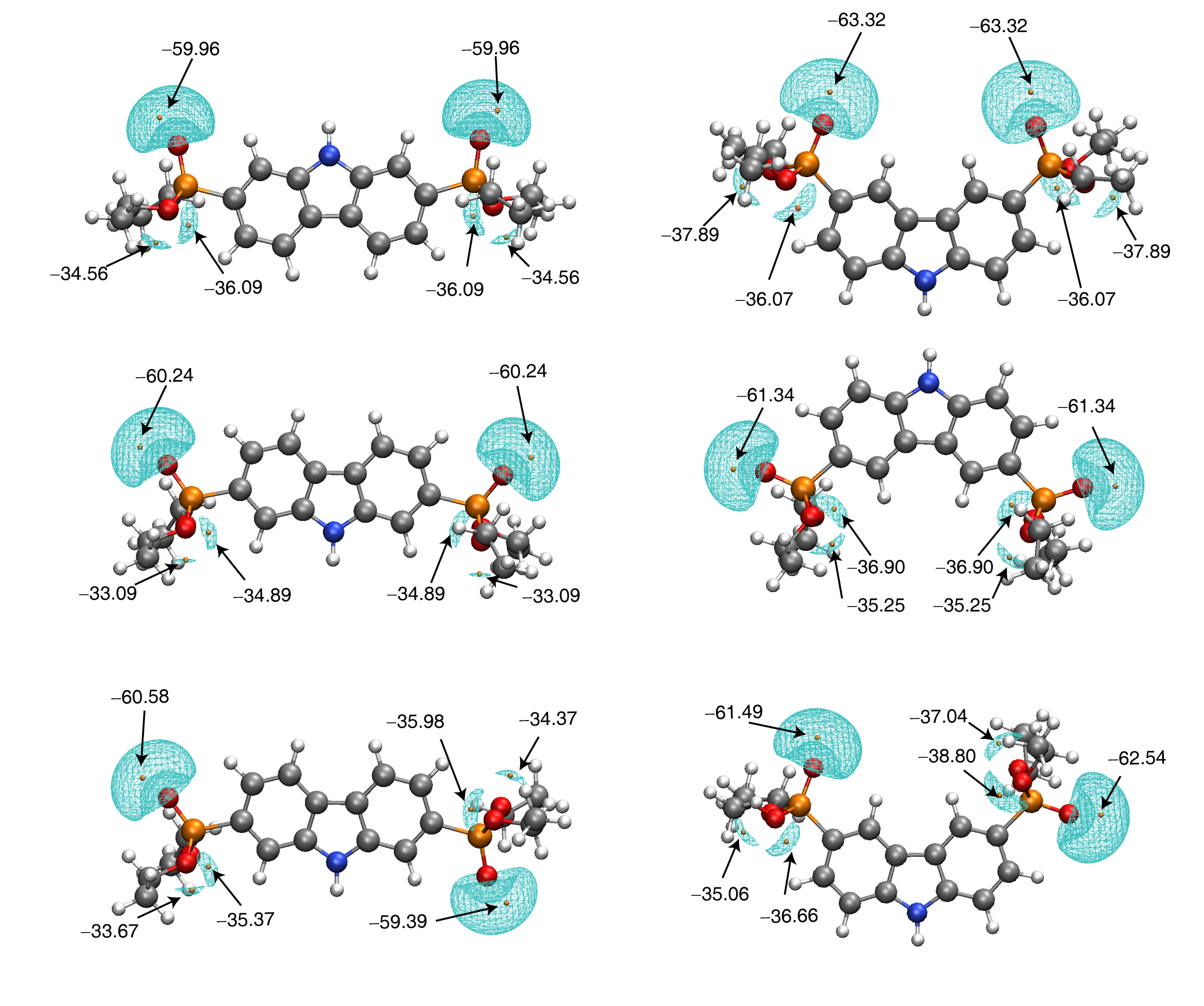


**Fig. S12** Electrostatic potential minima (ESPmin, shown as orange dots, values are labeled in kcal/mol) and isosurfaces (isovalue: -0.05 a.u., shown as cyan wireframes) for different 2,7-CzBP and 3,6-CzBP conformers, calculated at the B3LYP-D3(BJ)/def2-SVP level


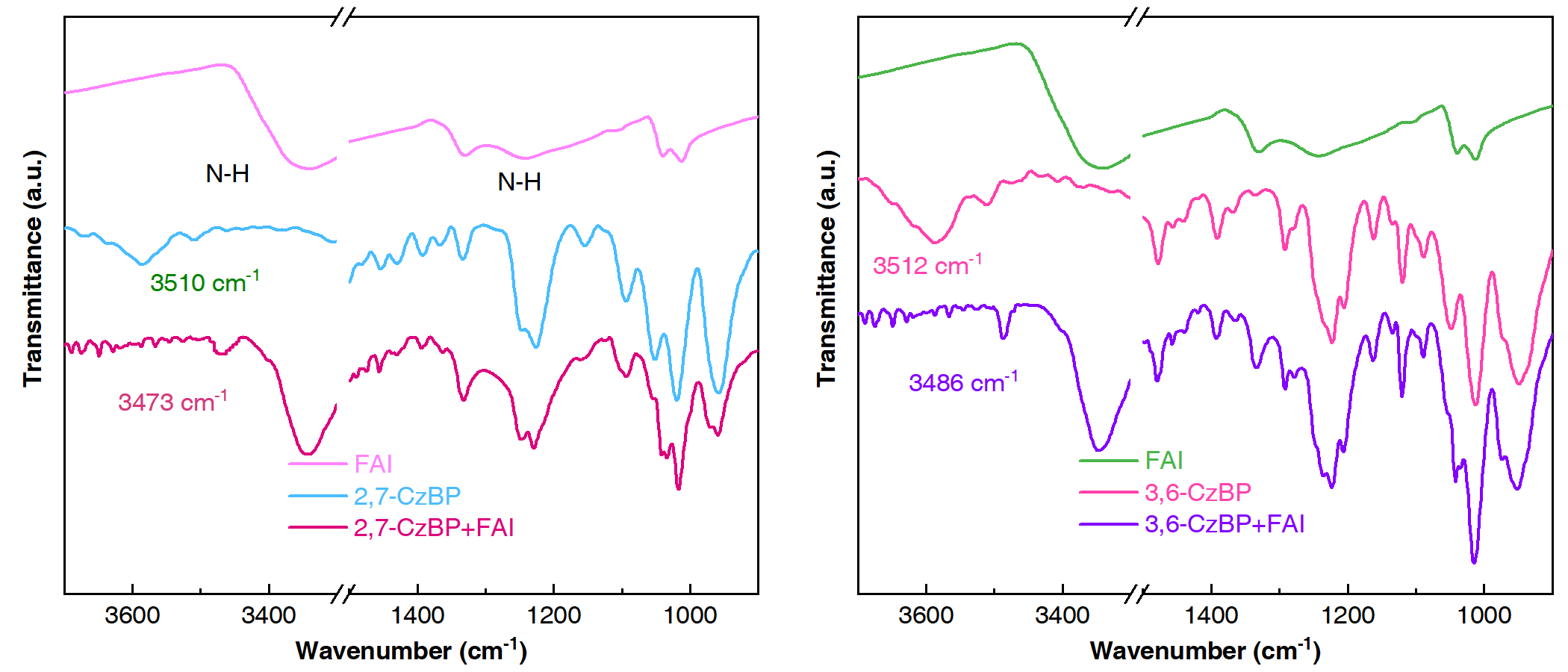


**Fig. S13** **a, b** FTIR spectra of FAI, 2,7-CzBP/FAI, and 3,6-CzBP/FAI. Perovskite film fabricated using perovskite precursors consisting of CzBP/FAI (1:1, mol%) in DMF: DMSO (8:1, v/v)


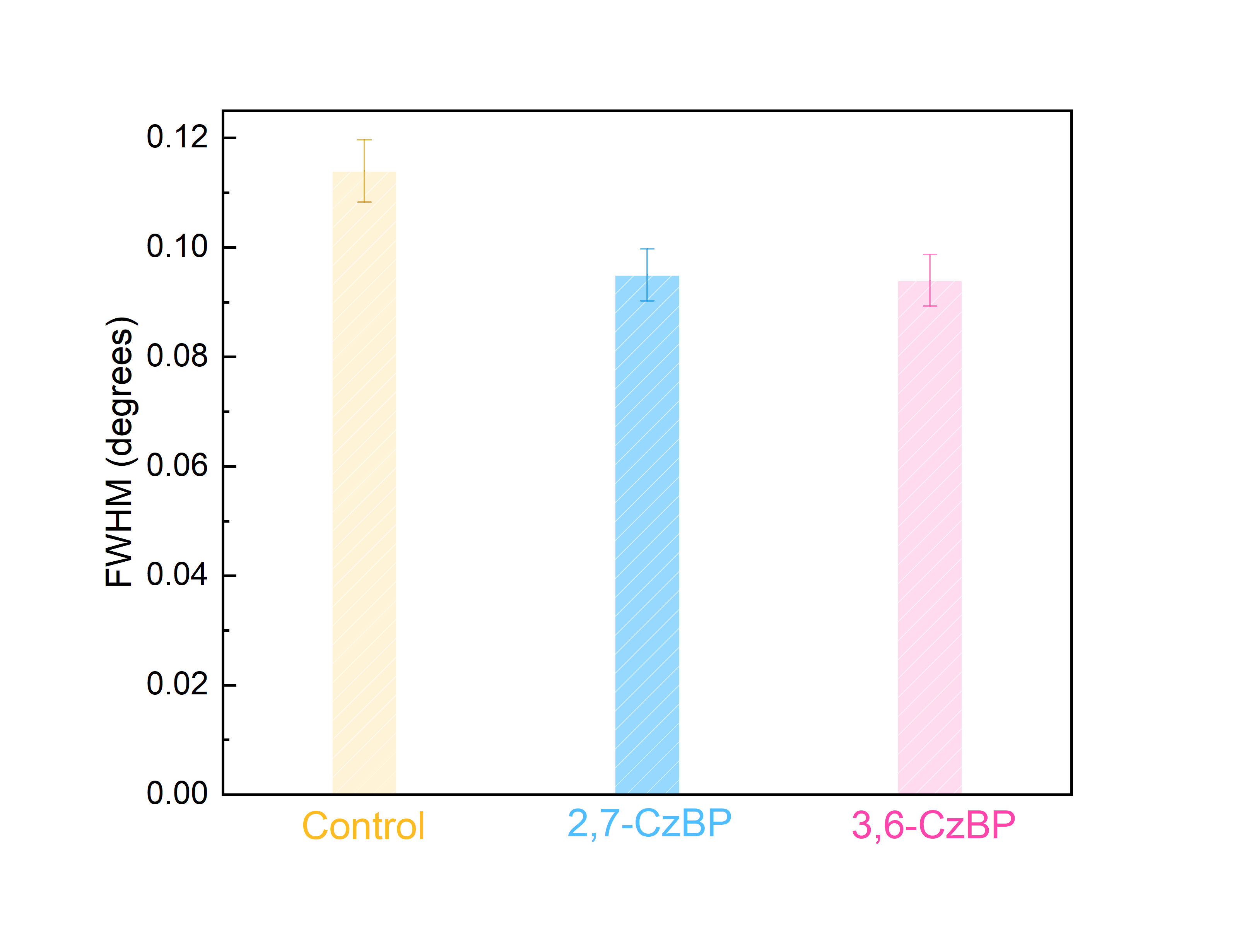


**Fig. S14** FWHMs of the control and CzBP-treated perovskite films


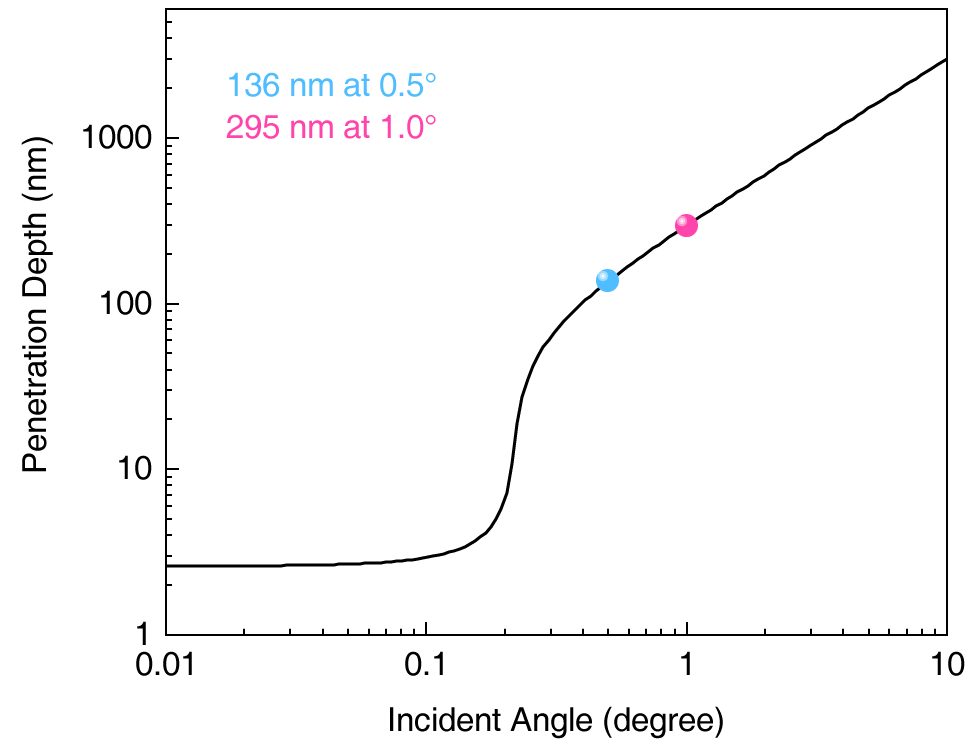


**Fig. S15** Penetration depths at incident angles of 0.5° and 1.0° for (FA_0.95_Cs_0.05_PbI_3_)_0.975_(MAPbBr_3_)_0.025_


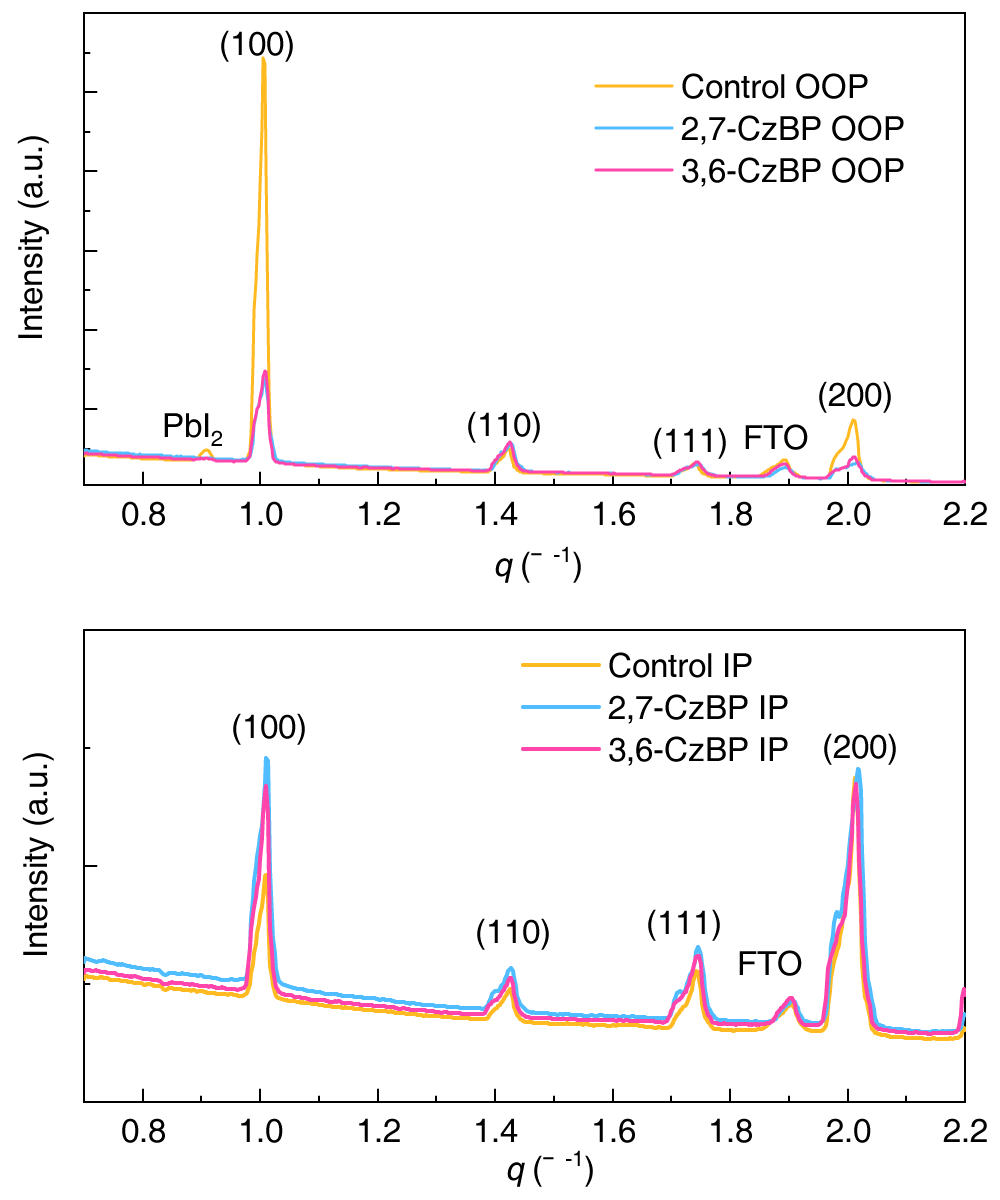


**Fig. S16** Out-of-plane (OOP) and in-plane (IP) cross-sectional integrated GIWAXS profiles at an incident angle of 0.5°


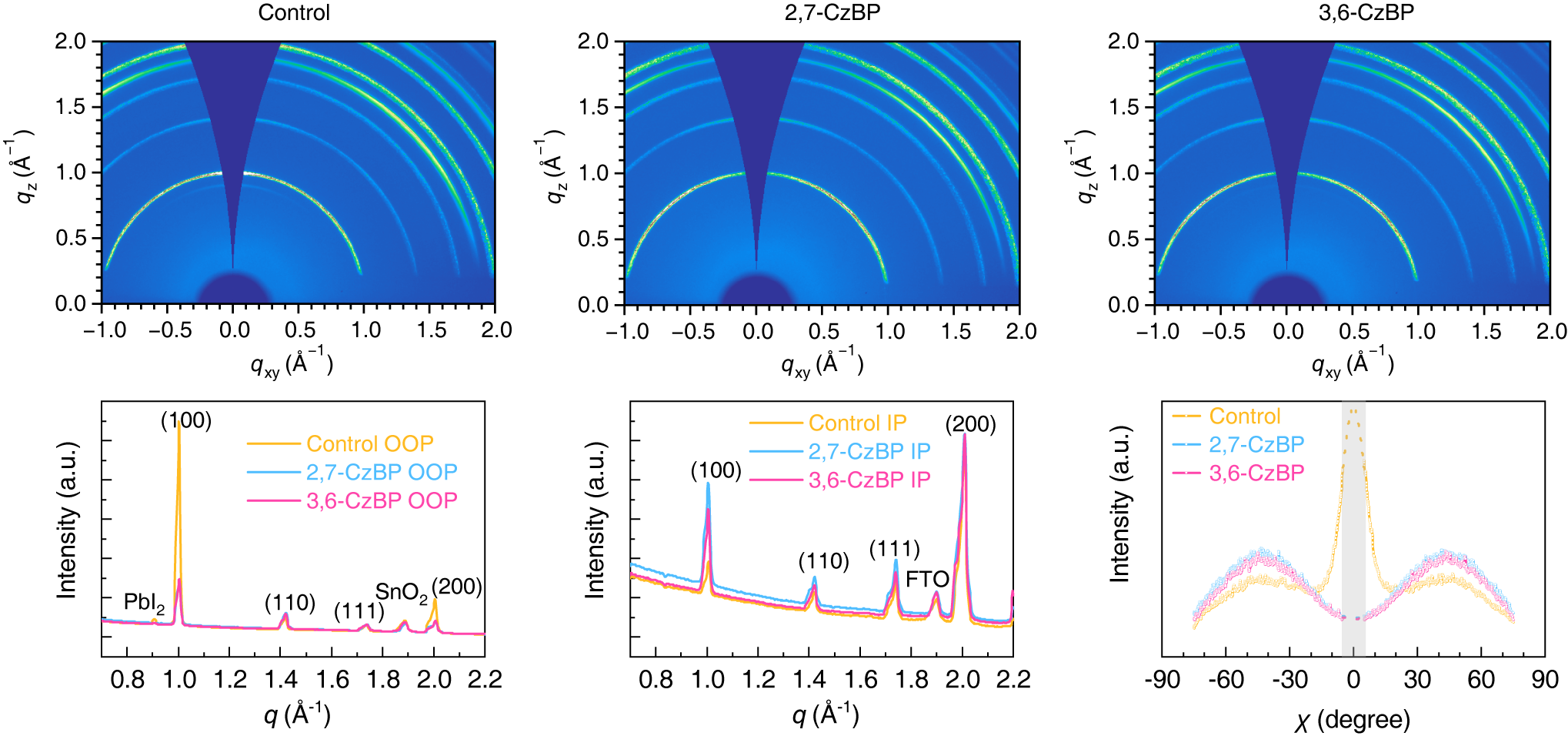


**Fig. S17** GIWAXS data, out-of-plane (OOP), in-plane (IP) cross-sectional integrated data, and I-χ curves at an incident angle of 1.0°


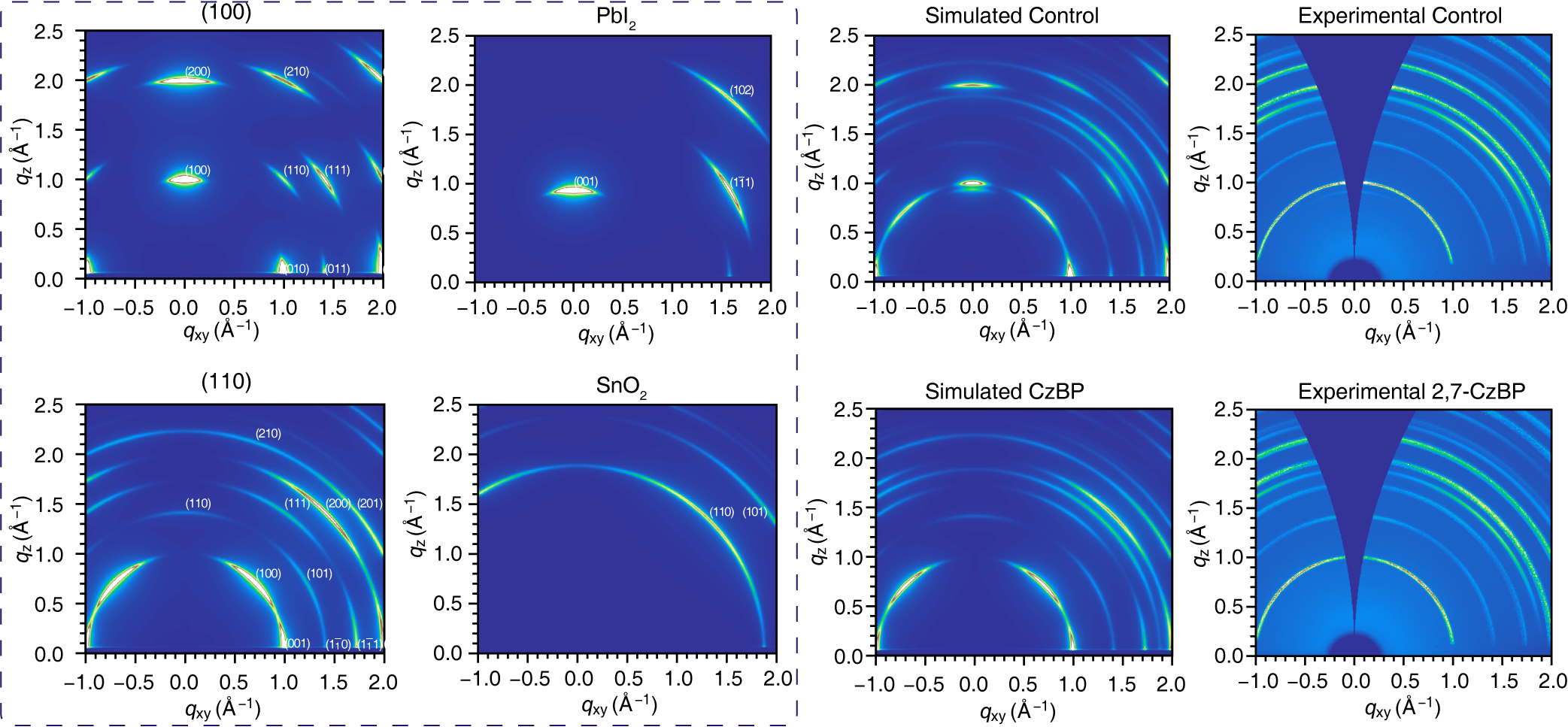


**Fig. S18** Simulated PVK (100), PVK (110), PbI_2_, SnO_2_ GIWAXS data at an incident angle of 0.5°. Simulated control and CzBP-treated data were combinations of the four simulated data


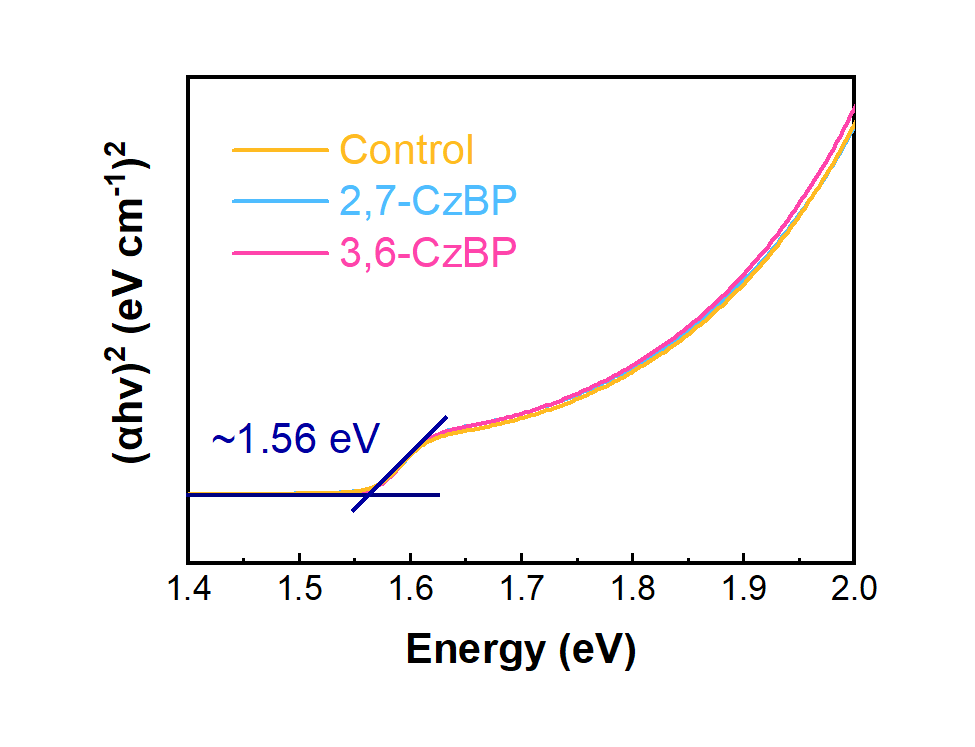


**Fig. S19** Tauc plots for control and CzBP-treated perovskite films


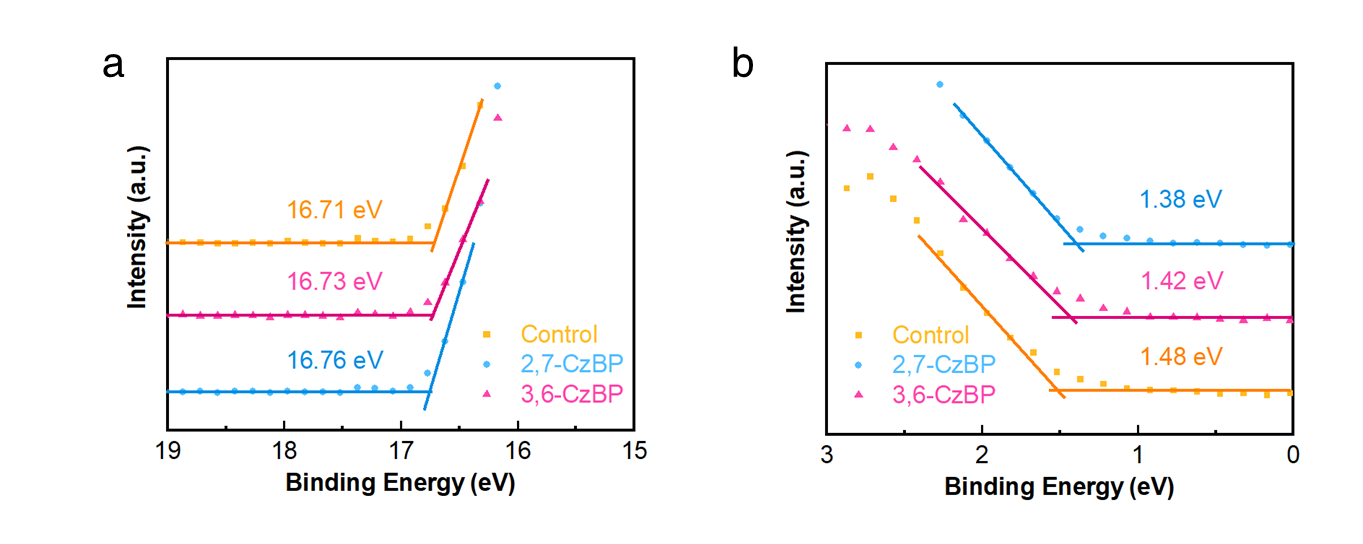


**Fig. S20** UPS spectra of perovskite films with and without CzBP treatment for **a** photoemission cutoff edges and **b** valence band spectra


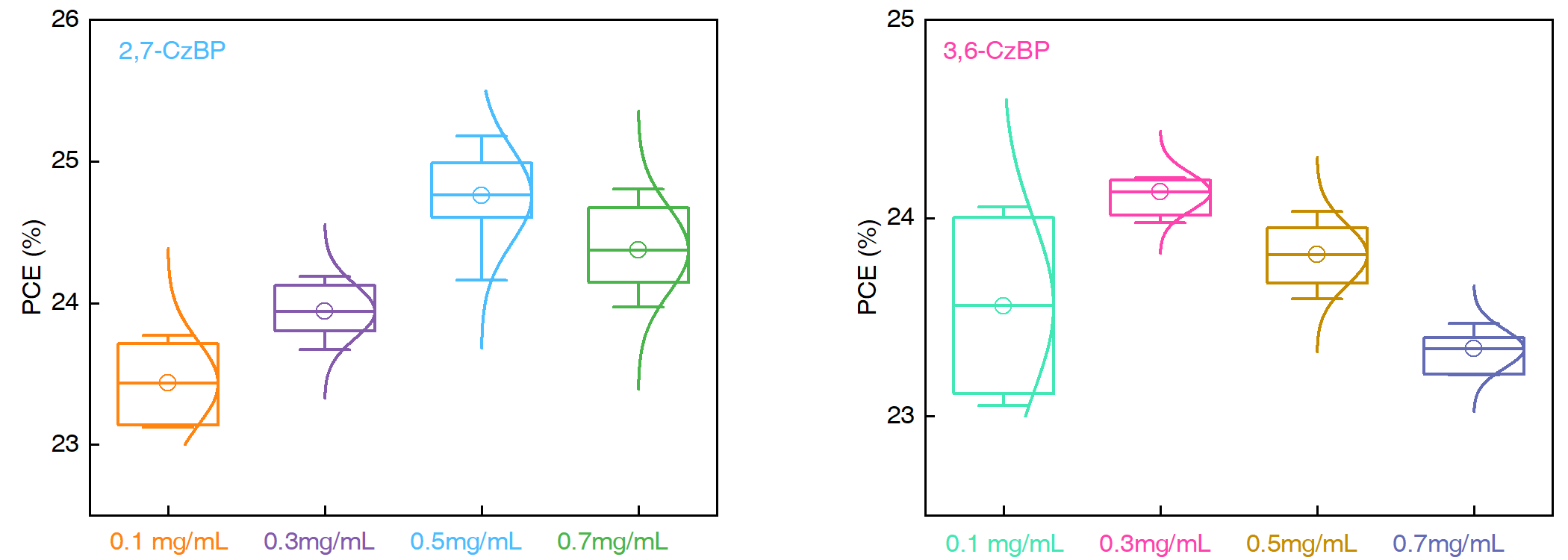


**Fig. S21** Comparison of devices with different concentrations of **a** 2,7-CzBP and **b** 3,6-CzBP in perovskite solutions


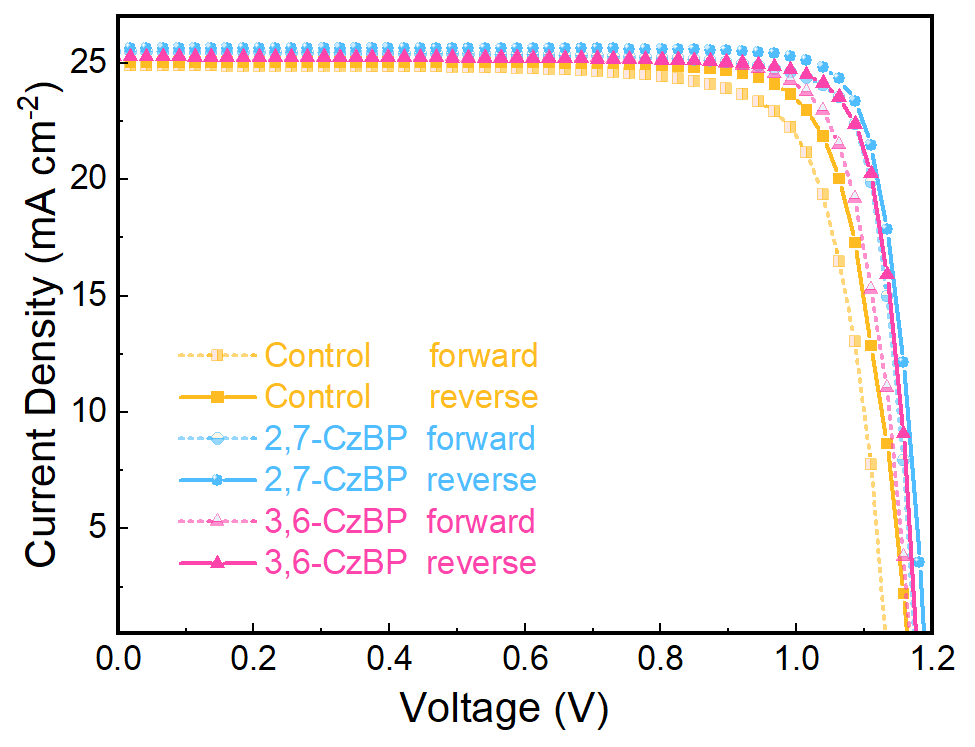


**Fig. S22** *J–V* curves of the PSCs with and without CzBP treatment in reverse and forward scanning directions


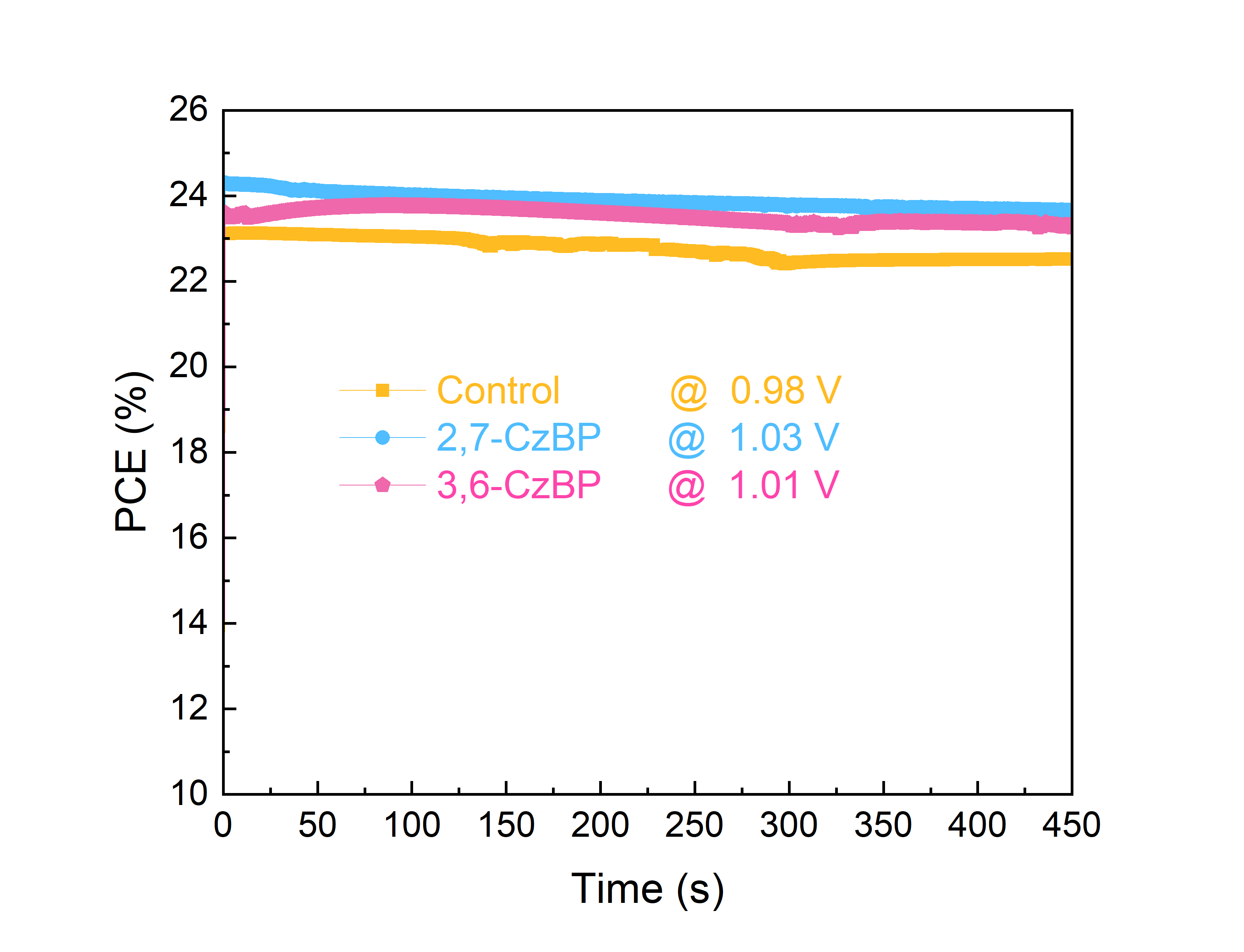


**Fig. S23** Stable output curves for devices with and without CzBP treatment


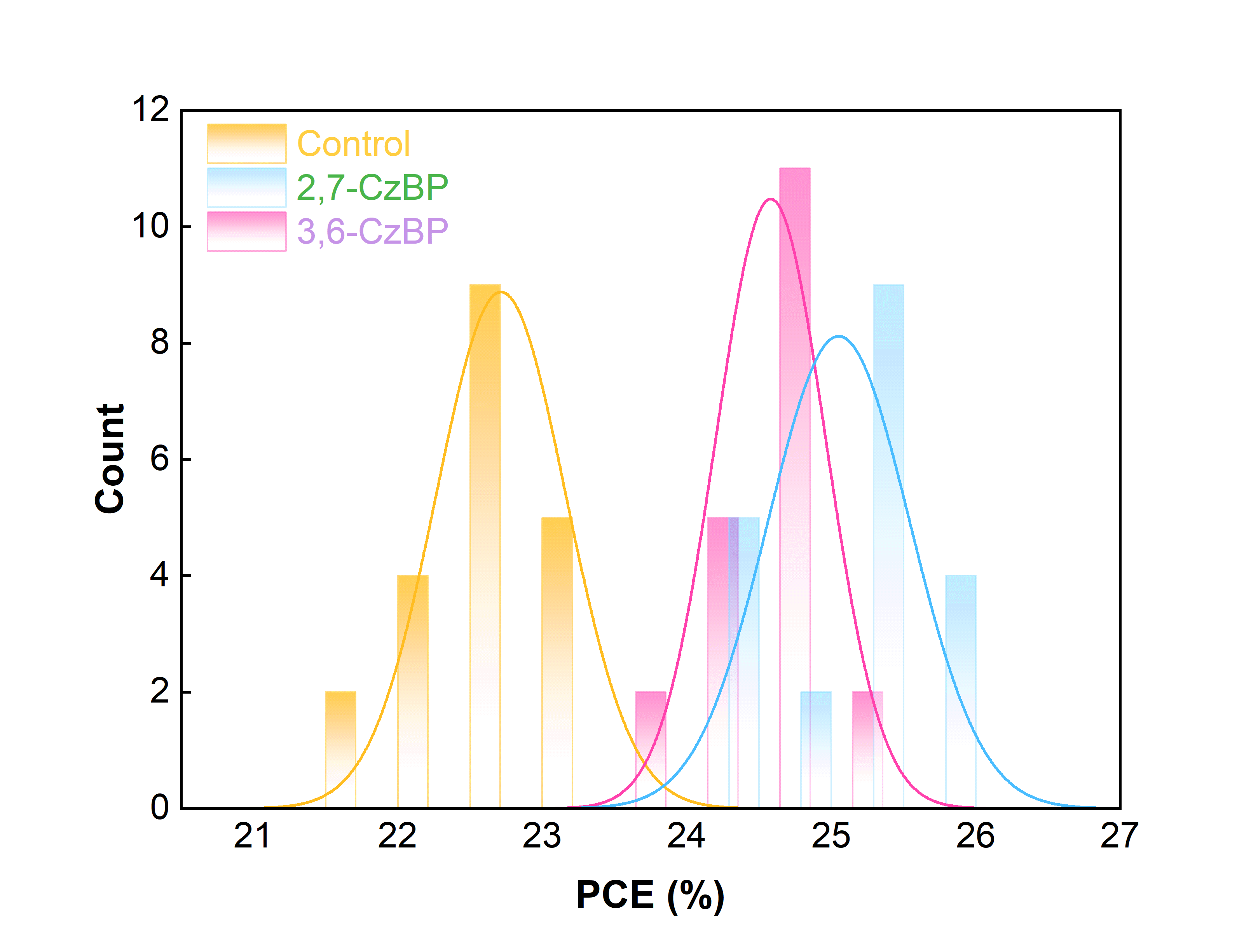


**Fig. S24** Histograms of PCEs for the control and CzBP-treated PSCs (60 devices)

**
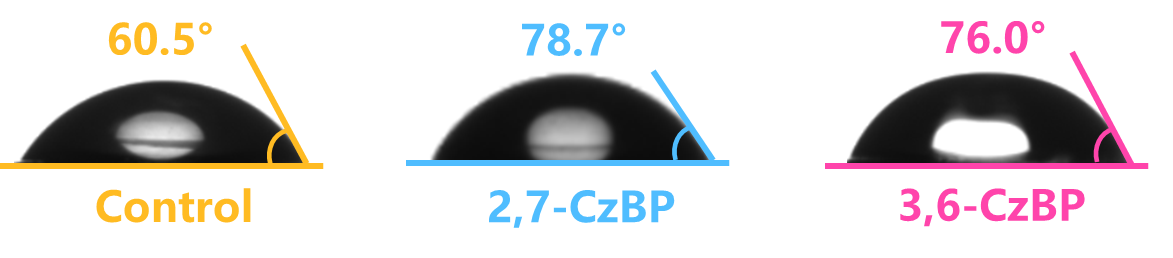
**

**Fig. S25** Contact angle measurement using water for the control and CzBP-treated perovskite films

**
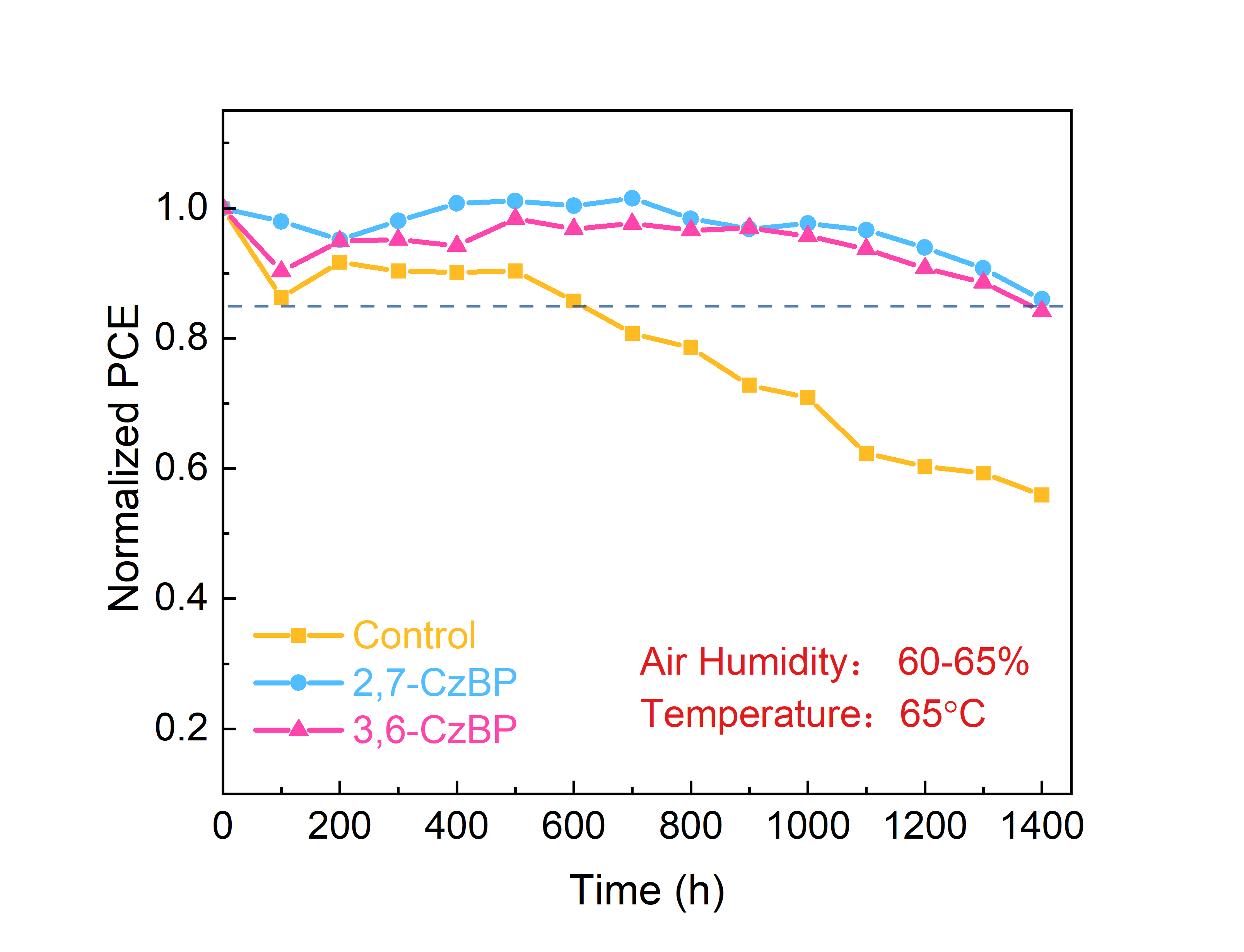
**

**Fig. S26** Thermal stability of unencapsulated devices with and without CzBP treatment at a temperature of 65 °C with RH of 60–65% in air

**
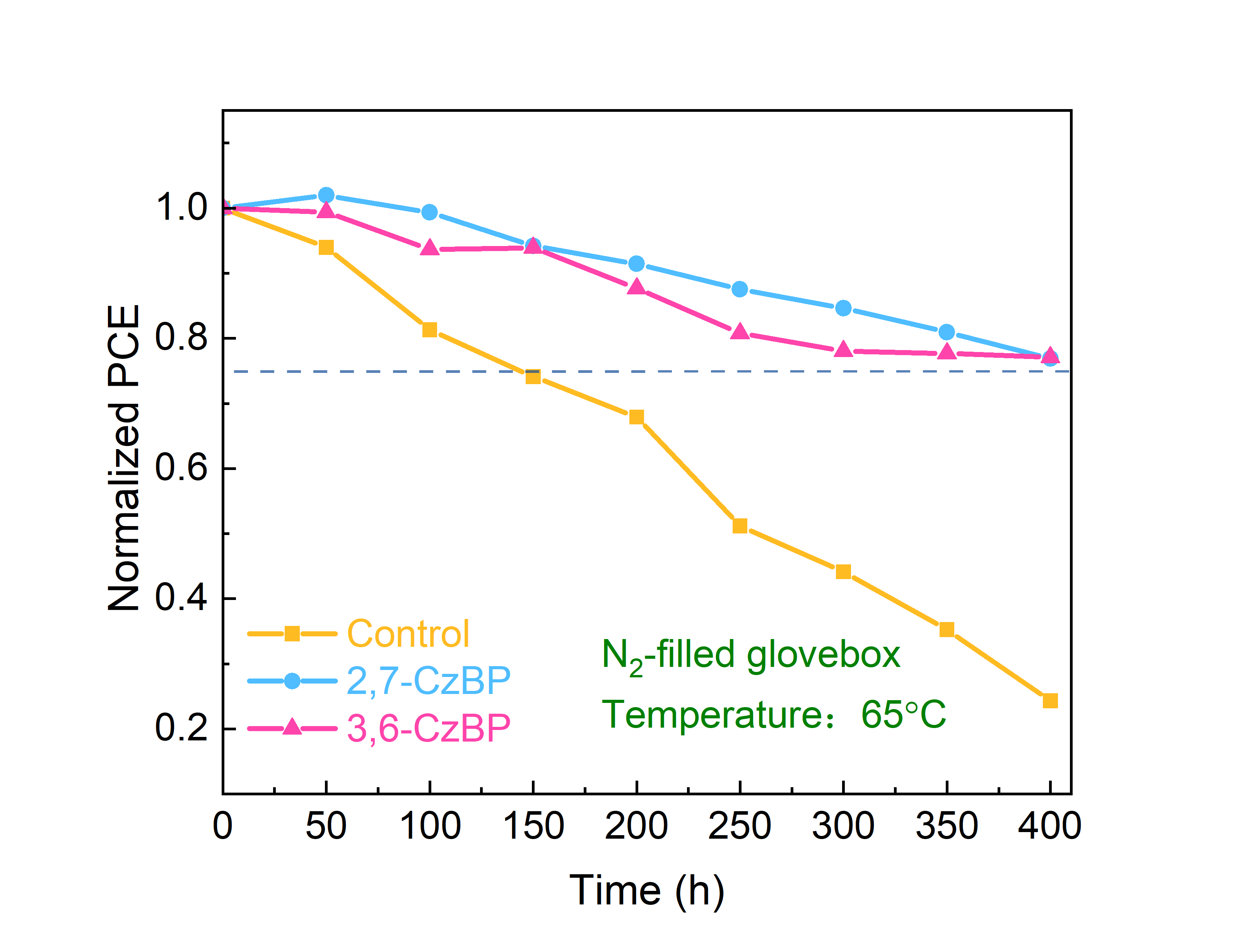
**

**Fig. S27** Thermal stability of unencapsulated devices with and without CzBP treatment at a temperature of 65 °C in a N_2_-filled glovebox


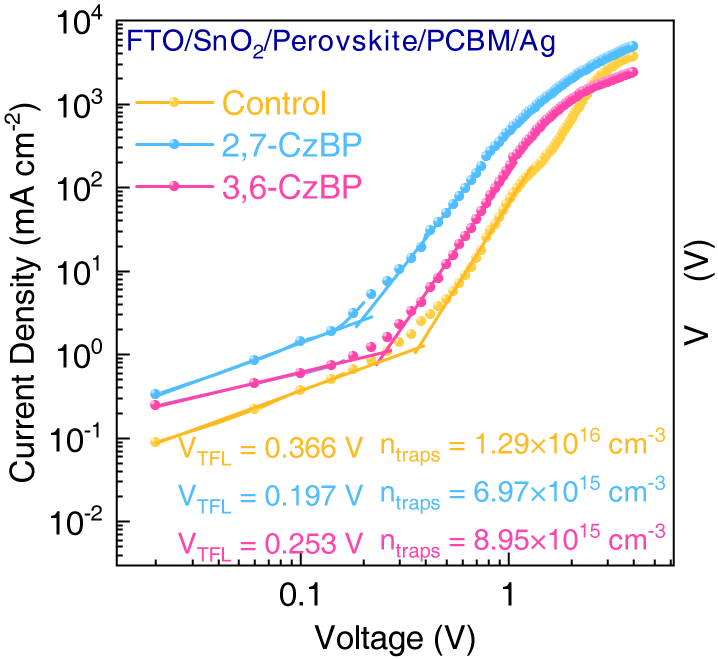


**Fig. S28** Trap density within the perovskite films for the electron-only devices (structure: FTO/SnO_2_/PVK (with and without CzBP addition)/PCBM/Ag). The control device exhibited a trap density (*N*_t_) of 1.29×10^16^ cm^−3^, while the 3,6-CzBP-treated device showed a reduced *N*_t_ of 8.95×10^15^ cm^−3^. Notably, the 2,7-CzBP-treated devices showed the lowest *N*_t_ of 6.97×10^15^ cm^−3^

**
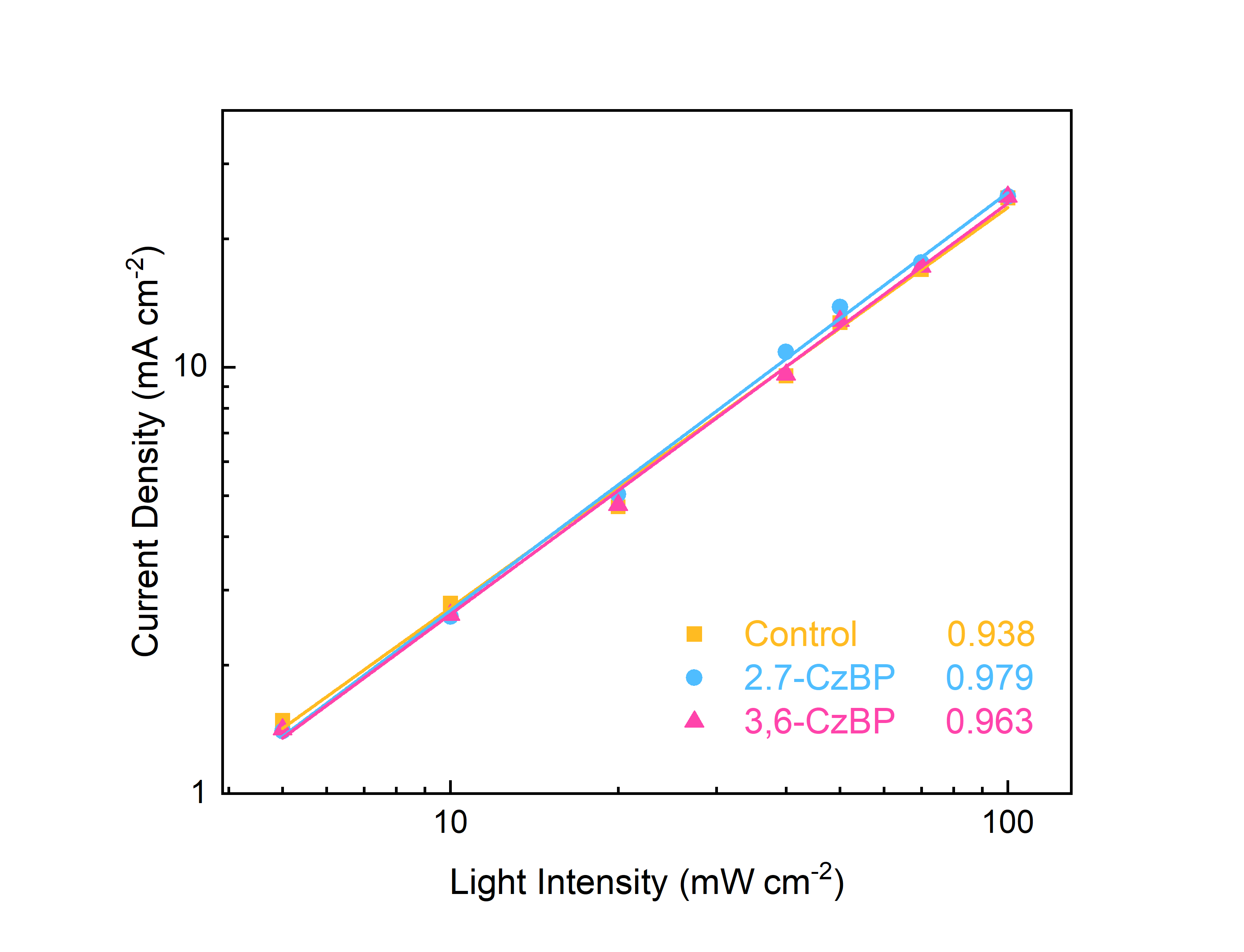
**

**Fig. S29** Dependence of *J*_SC_ on light intensity with and without CzBP treatment

**
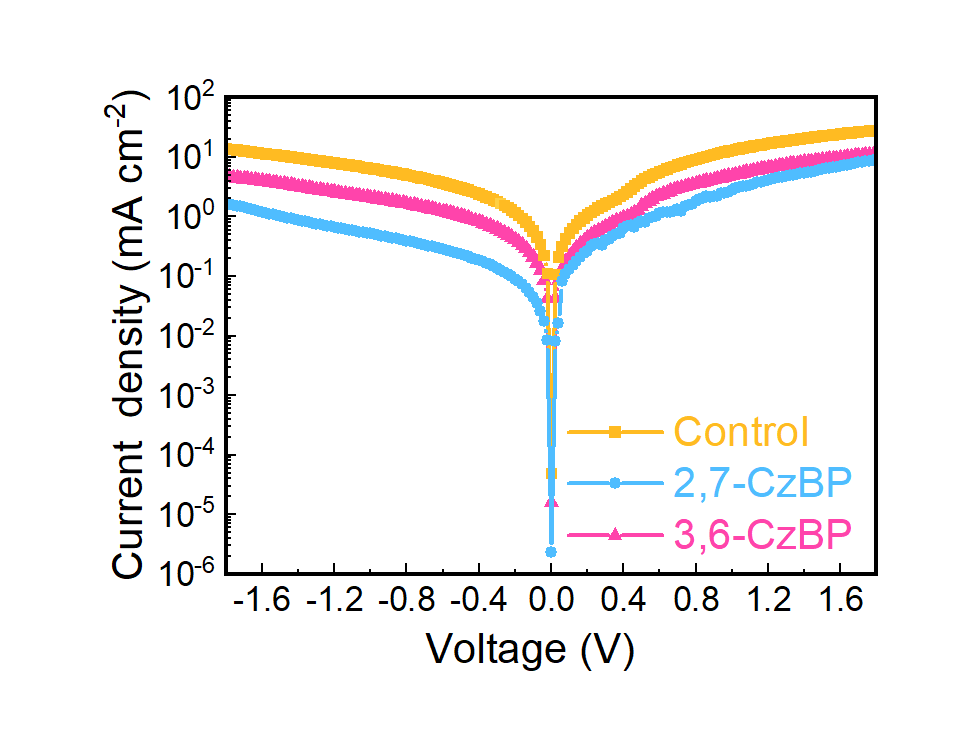
**

**Fig. S30** Dark *J-V* curve of the full PSCs with and without CzBP treatment

**
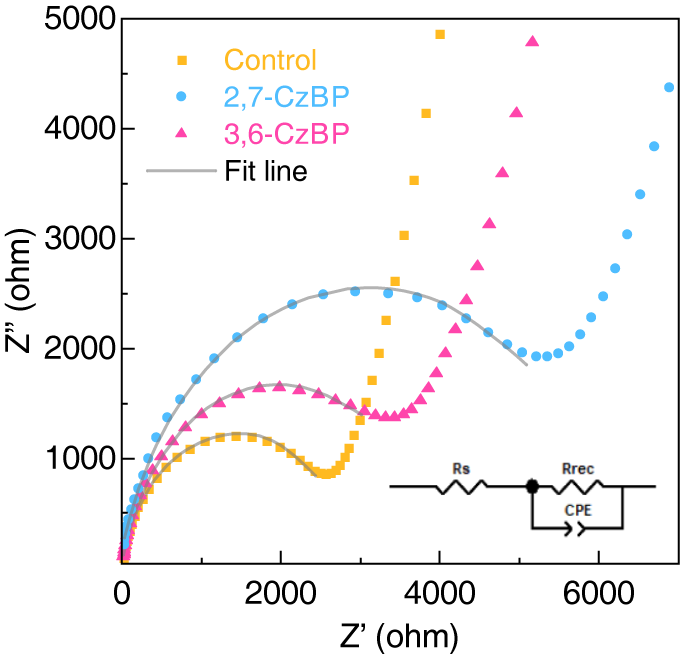
**

**Fig. S31** Electrochemical impedance spectroscopy (EIS) at a bias of 0.90 V under dark conditions

**
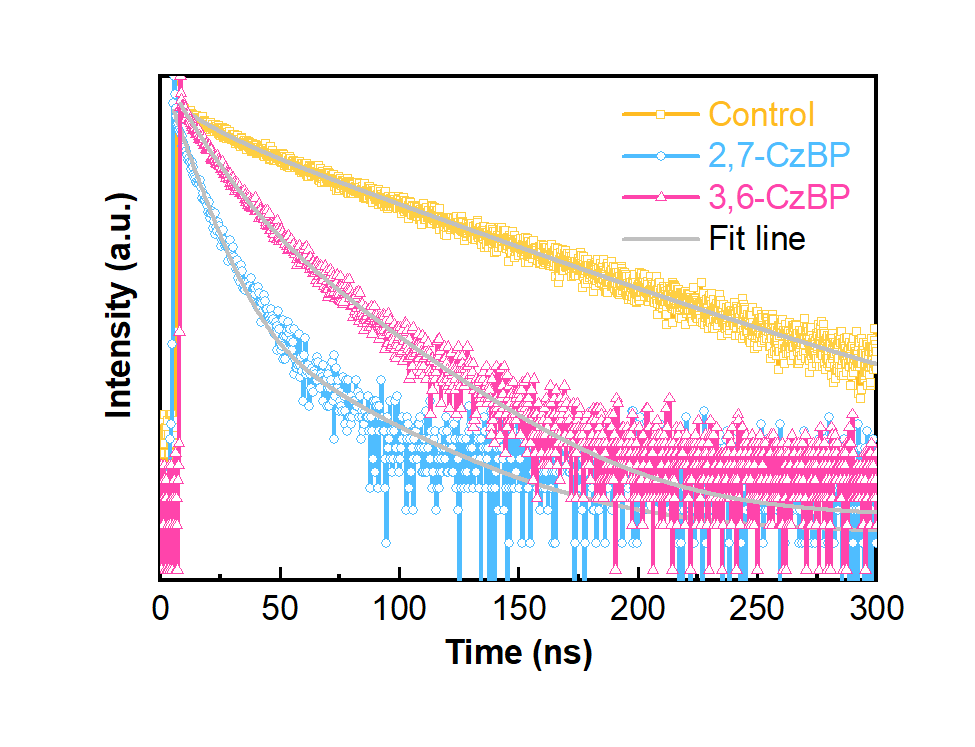
**

**Fig. S32** Time-resolved PL spectra of perovskite films with and without CzBP treatment. Condition: deposited on the glass substrate; configuration: glass/PVK/spiro-OMe TAD, excitation from the film side

**
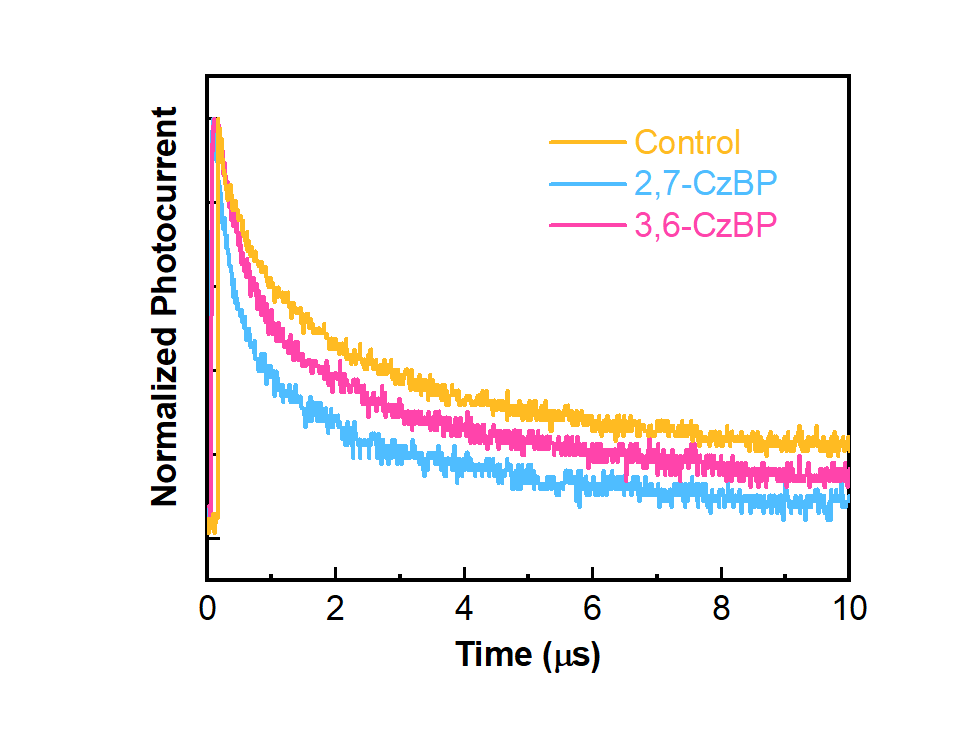
**

**Fig. S33** Transient photocurrent (TPC) at the short circuit of the full PSCs with and without CzBP treatment

**Table S1** TRPL lifetimes of the control and CzBP-treated films. The test structure was glass/perovskite

| Sample | *τ_1_* (ns) | *τ_2_* (ns) | *τ_ave_* (ns) |
| --- | --- | --- | --- |
| Control | 99.6 | 372.2 | 348.1 |
| 2,7-CzBP-addition | 148.6 | 600.5 | 560.6 |
| 3,6-CzBP-addition | 130.9 | 531.7 | 481.0 |

**Table S2** Photovoltaic parameters of the PSCs at optimized concentrations with and without CzBP treatment

| Sample |  | *V*_OC_ (V) | *J*_SC_ (mA cm^-2^) | FF (%) | PCE (%) |
| --- | --- | --- | --- | --- | --- |
| Control | Average  Maximum | 1.153 ± 0.014  1.164 | 25.33 ± 0.16  25.06 | 79.53 ± 1.29  80.43 | 22.81 ± 0.57  23.47 |
| 2,7-CzBP-addition | Average  Maximum | 1.183 ± 0.007  1.189 | 25.48 ± 0.17  25.66 | 82.97 ± 1.28  84.83 | 25.01 ± 0.56  25.88 |
| 3,6-CzBP-addition | Average  Maximum | 1.178 ± 0.007  1.177 | 25.45 ± 0.18  25.30 | 81.95 ± 1.33  84.24 | 24.58 ± 0.38  25.09 |

**Table S3** The parameters of PSCs under forward and reverse scans for the control and CzBP treated films

|  | *J*_SC_ (mA cm^−2^) | *V*_OC_ (V) | FF (%) | PCE (%) |
| --- | --- | --- | --- | --- |
| Control-R | 25.06 | 1.164 | 80.43 | 23.47 |
| Control-F | 24.99 | 1.133 | 78.33 | 22.17 |
| 2,7-CzBP-addition-R | 25.66 | 1.189 | 84.83 | 25.88 |
| 2,7-CzBP-addition-F | 25.49 | 1.175 | 83.66 | 25.07 |
| 3,6-CzBP-addition-R | 25.30 | 1.177 | 84.24 | 25.09 |
| 3,6-CzBP-addition-F | 25.27 | 1.168 | 81.70 | 24.12 |

**Table S4** Fitting results from the EIS of PSCs based on the control and CzBP-treated devices

|  | *R_S_* (Ω) | *R_tr_* (Ω) | *R_rec_* (Ω) |
| --- | --- | --- | --- |
| Control | 5.02 | 389 | 2909 |
| 2,7-CzBP-addition | 3.12 | 278 | 6270 |
| 3,6-CzBP-addition | 3.74 | 290 | 3974 |

**Table S5** TRPL lifetimes of the control and CzBP-treated films. The test structure was glass/perovskite/spiro-OMe TAD

| Sample | *τ_1_* (ns) | *τ_2_* (ns) | *τ_ave_* (ns) |
| --- | --- | --- | --- |
| Control | 19.1 | 75.3 | 69.7 |
| 2,7-CzBP-addition | 7.9 | 21.0 | 12.7 |
| 3,6-CzBP-addition | 10.7 | 30.1 | 22.7 |
